# Repetitive Levodopa Treatment Drives Cell Type-Specific Striatal Adaptations Associated With Progressive Dyskinesia in Parkinsonian Mice

**DOI:** 10.1101/2025.05.16.654598

**Published:** 2025-05-21

**Authors:** Rodrigo M. Paz, Michael B. Ryan, Pamela F. Marcott, Allison E. Girasole, Joe Faryean, Vincent Duong, Sadhana Sridhar, Alexandra B. Nelson

**Affiliations:** 1 Neuroscience Graduate Program, UCSF, San Francisco, CA 94158, USA; 2 Kavli Institute for Fundamental Neuroscience, UCSF, San Francisco, CA 94158, USA; 3 Weill Institute for Neurosciences, UCSF, San Francisco, CA 94158, USA; 4 Department of Neurology, UCSF, San Francisco, CA 94158, USA; 5 Aligning Science Across Parkinson’s (ASAP) Collaborative Research Network, Chevy Chase, MD 20815, USA; 6 Lead contact

## Abstract

The use of levodopa to manage Parkinson’s disease (PD) symptoms leads to levodopa-induced dyskinesia (LID) and other motor fluctuations, which worsen with disease progression and repeated treatment. Aberrant activity of striatal D1- and D2-expressing medium spiny neurons (D1-/D2-MSNs) underlies LID, but the mechanisms driving its progression remain unclear. Using the 6-OHDA mouse model of PD/LID, we combined *in vivo* and *ex vivo* recordings to isolate the effect of repeated treatment in LID worsening and other motor fluctuation-related phenotypes. We found that LID worsening is linked to potentiation of levodopa-evoked responses in both D1-/D2-MSNs, independent of changes in dopamine release or MSN intrinsic excitability. Instead, strengthening of glutamatergic synapses onto D1-MSNs emerged as a key driver. Moreover, we found changes in D2-MSN activity that specifically influenced LID duration, potentially contributing to motor fluctuations, which paralleled a reduction in D2R sensitivity. These findings reveal striatal adaptations contributing to worsening of levodopa-related complications.

## INTRODUCTION

In Parkinson’s disease (PD), the degeneration of dopamine neurons in the substantia nigra pars compacta leads to cardinal motor symptoms, including bradykinesia, rigidity and tremor^1,2^. Dopamine replacement therapy with levodopa remains the most effective symptomatic treatment in early stages^3,4^. However, as both the disease and levodopa treatment proceed, patients develop levodopa-induced dyskinesia (LID), characterized by abnormal involuntary movements, and other motor fluctuations, characterized by unpredictable changes in the duration of levodopa’s effects^5,6^. Over years of treatment, these motor complications gradually worsen, constraining treatment and significantly impairing quality of life^7,8^. Clinical research suggests that the progression of motor complications is influenced by three key factors: the extent of neurodegeneration, levodopa dosage, and treatment duration^7,9^. However, disentangling their individual contributions remains challenging, as these factors evolve simultaneously in patients. Despite its clinical significance, the mechanisms driving the progression of LID and motor fluctuations over time are poorly understood, hindering the development of effective therapeutic strategies.

Decades of research in people with PD as well as nonhuman primate and rodent models of PD have established the striatum as a critical site for LID pathophysiology^10–23^. The principal striatal neurons are GABAergic medium spiny neurons (MSNs), which can be classified into two major populations based on their dopamine receptor expression: D1 receptor-expressing (D1-MSNs) and D2 receptor-expressing (D2-MSNs)^24^. During movement, D1- and D2-MSNs are coactivated, which has been proposed to be essential for action selection, with D1-MSNs facilitating desired actions and D2-MSNs suppressing competing ones^25–27^. This model suggests that balanced activity of D1- and D2-MSNs is key for the execution of coordinated motor sequences^26,27^. LID can thus be conceptualized as a failure in action selection, where an imbalance between the activity of D1- and D2-MSNs leads to abnormal involuntary movements. Indeed, studies in mouse models of PD have demonstrated that levodopa administration induces opposing changes in the activity of these neurons, by increasing D1-MSN activity and decreasing D2-MSN activity^10,12,13,28,29^. Optogenetic and chemogenetic studies have further established a causal link between D1- and D2-MSN activity and LID expression^12,20,21,30–33^. However, very little is known about how levodopa-evoked D1- and D2-MSN responses evolve with repetitive levodopa treatment and how they contribute to the progression of LID and other motor fluctuations.

In the healthy brain, dopamine plays a key role in shaping action selection by modulating MSN function through changes in cellular excitability^34,35^ and glutamatergic synaptic plasticity^36–39^. For instance, transient increases and decreases in striatal dopamine in response to action outcomes can reinforce or suppress specific actions by strengthening or weakening corticostriatal synapses^37,40^. In PD, the chronic loss of dopamine disrupts this normal process, leading to marked changes in synaptic connectivity in target areas, such as the striatum^14,41^. In late-stage PD with LID, the relationship between dopamine and action outcomes is further disrupted as levodopa leads to a non-physiologic spatiotemporal pattern of striatal dopamine release. These sustained increases and decreases in dopamine signaling have been shown to drive a range of cellular adaptations in MSNs, including morphological remodeling, alterations in intrinsic excitability and changes in synaptic connectivity^14–17,42–45^. Despite these insights, previous research has primarily examined striatal adaptations once LID has already progressed, overlooking the changes that unfold alongside the increase in LID severity. As a result, it is unclear which adaptations drive LID worsening and which are compensatory. We propose that chronic dopamine depletion drives homeostatic but also maladaptive changes in corticostriatal and striatal circuitry, which lead to progressive vulnerability to LID in PD. Compounding this problem is the potential effect of levodopa on striatal plasticity, which may contribute to worsening of LID and motor fluctuations, such as rapid wearing off, over treatment.

Here, we hypothesized that repeated cycles of levodopa-driven dopamine fluctuations promote LID progression by hijacking endogenous mechanisms for striatal synaptic plasticity, leading to changes in the balance between D1- and D2-MSN activity. To test this hypothesis, we used the 6-hydroxydopamine (6-OHDA) mouse model of PD/LID to isolate the effects of levodopa treatment duration on LID progression and identify key timepoints during treatment associated with worsening of dyskinesia and other motor fluctuation-like phenotypes. Using fiber photometry and single-unit electrophysiology, we tracked changes in D1- and D2-MSN activity, as well as dopamine release, across these critical stages of LID progression. Our findings reveal that repeated levodopa administration is likely to exacerbate LID by amplifying levodopa-induced increases in D1-MSN activity and decreases in D2-MSN activity. Furthermore, using *ex vivo* slice electrophysiology, we demonstrate that LID worsening is associated with cell type-specific potentiation of glutamatergic transmission. Finally, we show that dyskinesia resolves more quickly over the treatment course, potentially reflecting shared mechanisms with the “wearing off” phenomenon seen during chronic treatment. This more rapid resolution of LID is accompanied by altered D2-MSN activity and reduced D2 receptor sensitivity.

## RESULTS

### Repetitive levodopa treatment worsens LID severity and speeds its onset and offset.

**Figure 1.**
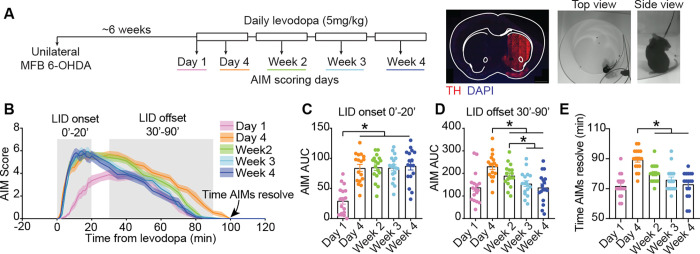
Repetitive levodopa treatment worsens LID severity and speeds its onset and offset. **(A)** Experimental approach. Left: Timeline. Middle: Representative coronal section showing TH loss in the hemisphere ipsilateral to 6-OHDA injection. Right: A dyskinetic mouse seen from a top and side view. **(B)** Average abnormal involuntary movement (AIM) score after levodopa injection (5 mg/kg, IP) across treatment timepoints. **(C-D)** Area under the curve (AUC) of AIM score during LID onset (C), and offset (D) periods. **(E)** Time to AIM resolution after levodopa injection. [C-E: RM one-way ANOVA, p<0.0001, post-hoc: *p<0.05]. Data shown as mean±SEM. N=17 mice.

To examine how repetitive levodopa administration contributes to LID progression, we used the 6-OHDA mouse model of PD. The neurotoxin 6-OHDA was injected unilaterally in the medial forebrain bundle (MFB), resulting in severe unilateral loss of dopamine neurons (Figure 1A). After several weeks of recovery, mice were treated with levodopa-benserazide (5 mg/kg-2.5 mg/kg, by daily IP injection) for four weeks. In response to levodopa, mice increased their overall velocity (Figure S1A) and developed dyskinesia, which was scored using the abnormal involuntary movement (AIM) scale^46^ at several timepoints across the treatment session (Figure 1A–B). The time course and severity of dyskinesia changed over the course of treatment (Figure 1B), consistent with work by other investigators^19,46^. Most notably, the severity of dyskinesia increased between day 1 and all subsequent days. In addition, the total duration of dyskinesia increased between day 1 and day 4, after which it declined again. To quantify these changes, we focused our analysis on two distinct time windows: **LID onset** (the first 20 minutes after levodopa injection) and **LID offset** (30 to 90 minutes post-injection) (Figure 1B). During the LID onset phase, dyskinesia increased from day 1 to day 4 with no further changes thereafter (Figure 1B–C). In contrast, during the LID offset phase, dyskinesia resolved faster between day 4 and week 4 (Figure 1B, D–E). Levodopa-induced increases in movement velocity also changed over time during both onset and offset phases (Figure S1A–C). During the onset phase, increases in movement velocity were markedly attenuated from day 1 to day 4, which may reflect the fact that severe dyskinesia impairs normal movement (Figure S1A–B). In contrast, during the offset phase, movement velocity gradually increased across weeks, though the total period of increased velocity was consistently about 120 minutes (Figure S1C). Collectively, these findings indicate that repeated levodopa administration drives two major changes: (1) a more rapid onset and greater severity of LID, and (2) more rapid LID offset.

### Changes in striatal activity parallel alterations in dyskinesia during repetitive levodopa treatment.

**Figure 2.**
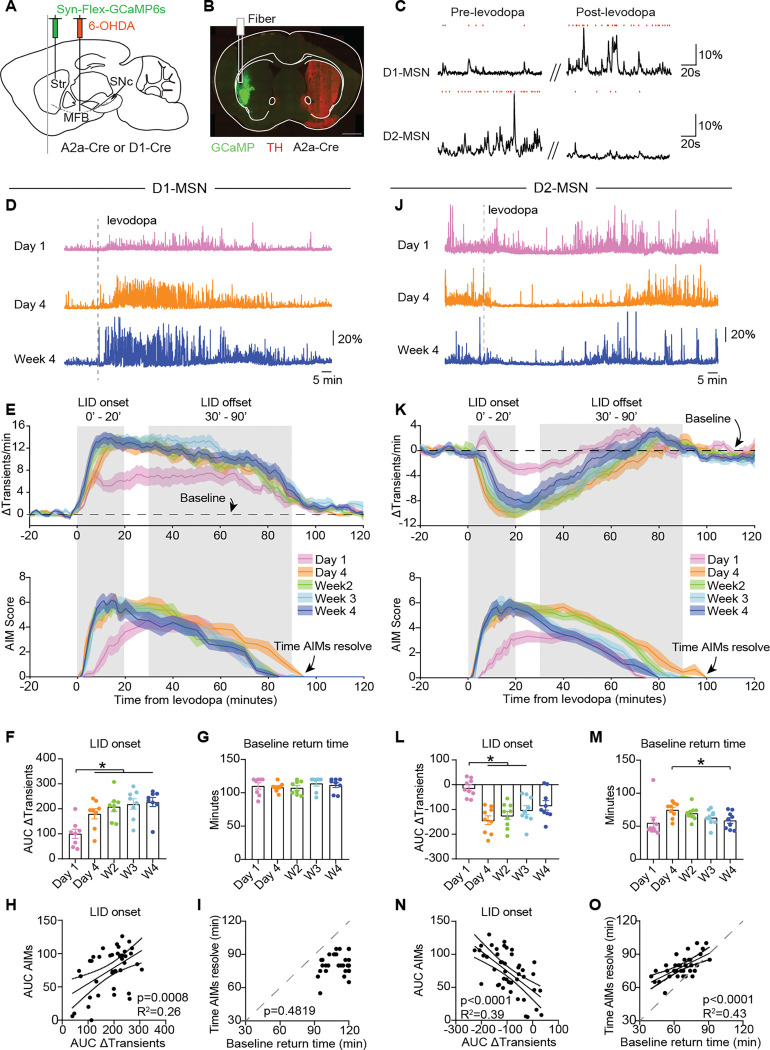
Changes in striatal activity parallel alterations in dyskinesia during repetitive levodopa treatment. **(A)** Schematic showing injection of AAV encoding Flex-GCaMP6s into the dorsolateral striatum (Str) and 6-OHDA into the ipsilateral medial forebrain bundle (MFB) of D1-Cre or A2A-Cre mice**. (B)** Representative postmortem coronal section showing GCaMP6s expression and TH loss, as well as the fiber track. **(C)** Representative short sweeps from GCaMP6s fiber photometry recordings (ΔF/F) from D1- and D2-MSN before and after levodopa, with red ticks marking GCaMP transients. **(D-I)** Analysis of fiber photometry data from D1-MSNs. **(D)** Representative fiber photometry recordings (ΔF/F) from D1-MSN across treatment timepoints. Each trace depicts an entire 140-minute session. **(E)** Top: Change in the rate of GCaMP6s transients relative to baseline after IP levodopa across treatment timepoints. Bottom: AIM scores for D1-Cre mice. **(F)** Area under the curve (AUC) of Δtransients during LID onset period. [RM one-way ANOVA, p=0.0003, post-hoc: *p<0.05]. **(G)** Time for transient frequency to return to baseline rate. [RM one-way ANOVA, ns]. **(H)** Correlation of Δtransients with AIMs during LID onset period. **(I)** Correlation between the time to resolution AIMs and the time to return of transient frequency to baseline (time). **(J-O)** Analysis of fiber photometry data from D2-MSNs. **(J)** Representative fiber photometry recordings (ΔF/F) from D2-MSN across treatment timepoints. **(K)** Top: Change in the rate of GCaMP6s transients relative to baseline after IP levodopa across treatment timepoints. Bottom: AIM scores for A2a-Cre mice. **(L)** Average AUC of Δtransients during LID onset period. [RM one-way ANOVA, p=0.0001, post-hoc: *p<0.05]. **(M)** Time for transient frequency to return to baseline. [RM one-way ANOVA, p=0.0047, post-hoc: *p<0.05]. **(N)** Correlation of Δtransients with AIMs during LID onset period. **(O)** Correlation between the resolution of AIMs (time) and the return of transient frequency to baseline (time). N=8 D1-Cre and N=9 A2a-Cre. Each dot is a session of one mouse. Data shown as mean±SEM (E-G; K-M).

Neural activity in the striatum has been causally linked to LID^12,20,30^. However, it is unclear how striatal activity evolves with repetitive levodopa administration, and how this activity may contribute to the evolution of dyskinesia over time. To fill this gap, we measured the activity of the two canonical striatal projection neuron types, D1-MSNs and D2-MSNs, over the course of chronic levodopa treatment. We performed cell type-specific fiber photometry with the genetically-encoded calcium indicator GCaMP6s. In 6-OHDA-treated animals, we injected an AAV encoding Cre-dependent GCaMP6s into the dorsolateral striatum (DLS), targeting expression to D1- or D2-MSNs (Figure 2A). Mice were implanted with a 400μm optic fiber in DLS and recorded at different timepoints over the course of four weeks of levodopa treatment (Figure 2B). In these recordings, we observed transient elevations of the fluorescence signal, which could be quantified over time and across sessions. Consistent with previous studies using both single-unit electrophysiology and GCaMP calcium imaging^10,28^, in single sessions, levodopa acutely increased D1-MSN activity and decreased D2-MSN activity (Figure 2C).

We next compared the response of each striatal subpopulation to levodopa across 1 month of repeated administration. Given the close linkage of increased D1-MSN activity to LID^10,12,13,21,28–30,47,48^, we hypothesized that increases in D1-MSN activity across sessions might parallel the worsening of LID over time. We compared D1-MSN responses to levodopa, as measured by GCaMP, at day 1, day 4, and weeks 2–4. We measured these responses by comparing the fluorescence transient frequency (Figure 2D–E). Importantly, baseline transient rates (pre-levodopa administration) were unchanged by repetitive treatment (Figure S2A). Paralleling the increase in dyskinesia severity between day 1 and day 4 during the onset phase, we observed greater elevations in D1-MSN activity during the LID onset phase from day 4 onward (Figure 2E–F). Between day 4 and week 4, we observed a more rapid offset and faster resolution of LID, but this behavioral change was not paralleled by any change in D1-MSN activity in the LID offset phase (Figure S2B; Figure 2E). Moreover, the time required for D1-MSN activity to return to pre-levodopa baseline levels remained constant across treatment timepoints (Figure 2G). To further examine the link between D1-MSN activity and AIM scores during LID onset and offset phases, we correlated the area under the curve (AUC) of the AIM score with the change in transient frequency. Remarkably, D1-MSN activity showed a robust correlation with AIM scores during the LID onset, but not during the offset phase (Figure 2H; Figure S2C). In line with this result, the time required for D1-MSN activity to return to pre-levodopa baseline levels did not correlate with the time that AIMs resolved (Figure 2I). Together, these findings suggest that D1-MSN activity may contribute to the worsening of dyskinesia severity, but may not explain why LID resolves more rapidly at later timepoints in treatment.

D2-MSNs have also been closely linked to LID, as chemogenetic or optogenetic activation attenuates LID severity^20,30^. We hypothesized that changes in LID severity and kinetics might also be mediated by changes in D2-MSN responses to levodopa over time. While D2-MSN activity was reduced by levodopa at all time points, this reduction was more pronounced by day 4 of treatment (Figure 2J–L). This reduction was prominent during the LID onset phase, paralleling the increase in LID severity from day 1 to day 4 (Figure 2K–L), with no changes in baseline D2-MSNs GCaMP transient frequency (Figure S2D). Moreover, the recovery of D2-MSN activity was more rapid during the offset phase from day 4 to week 4 (Figure 2M; Figure S2E), which paralleled the more rapid resolution of AIMs (Figure 2K). Indeed, D2-MSN activity was strongly correlated with AIM scores for both LID onset and offset periods (Figure 2N; Figure S2F), and the time required for D2-MSN activity to return to pre-levodopa baseline levels showed robust correlations with the time required for AIMs to resolve (Figure 2O). Importantly, we obtained identical results when using ΔF/F amplitudes, instead of transient frequency, as a proxy of neural activity, both for D1- and D2-MSNs (Figure S3). These results indicate that alterations in D2-MSN responses to levodopa may contribute to changes in the onset and offset of LID over the course of treatment.

In sum, our data show that during the LID onset phase, both D1- and D2-MSN show strong correlations with dyskinesia severity. In contrast, during the LID offset phase, D1-MSN activity evolves slowly and remains unchanged between day 4 and week 4, despite pronounced behavioral changes: LID resolves more rapidly (Figure 1B), and locomotor velocity increases (Figure S1A). These findings suggest that D2-MSNs play a critical role in shaping behavior during this post-levodopa period. Specifically, for similar levels of D1-MSN activation, D2-MSNs may determine whether mice express dyskinesia or instead exhibit increased locomotor velocity consistent with a therapeutic effect.

### Repetitive levodopa potentiates the responses of single striatal neurons, without changes in dopamine release.

**Figure 3.**
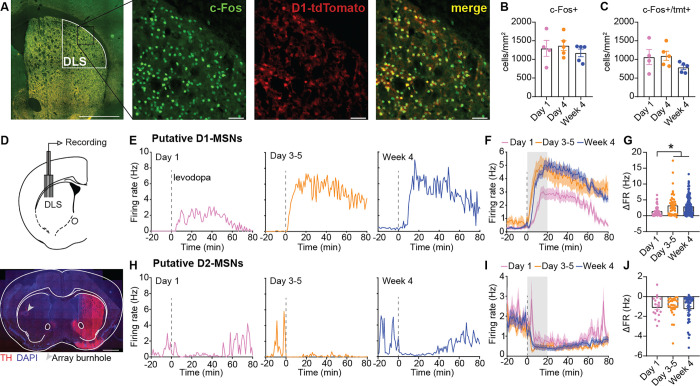
Repetitive levodopa treatments potentiate single neuron responses. **(A-C)** Drd1a-tdTomato mice were injected with unilateral 6-OHDA, after which they were treated with IP levodopa (5 mg/kg) for 1 day, 4 days, or 4 weeks. Animals were sacrificed 2 hours after the last levodopa treatment for postmortem c-Fos immunohistochemistry. **(A)** Representative coronal section showing D1-tdTomato (D1-MSNs) and c-Fos immunofluorescence in the dorsolateral striatum (DLS). Inset: High magnification images of c-Fos, D1-tdTomato, and merged image. **(B-C)** Density of c-Fos+ cells (B) and c-Fos+/D1+ (colocalized) cells (C) across treatment timepoints. [Kruskal-Wallis, ns]. Each dot represents one mouse (N=4–5). **(D)** Schematic of multielectrode array implant in DLS (top) and postmortem histology showing recording site with electrolytic lesion (bottom). **(E-F)** Representative single units (E) and pooled single unit firing rates (F) of putative D1-MSN on days 1, 4 and week 4 aligned to levodopa injection. **(G)** Change in firing rates between baseline and LID onset period (gray bar in F) for putative D1-MSN. [Kruskal-Wallis, p=0.0009, post-hoc: *p<0.05]. Day 1: n=55, N=10; Day 4: n=48, N=10; Week 4: n=157, N=14. **(H-I)** Representative single units (H) and pooled single unit firing rates (I) of putative D2-MSN on days 1, 4 and week 4. **(J)** Change in firing rates between baseline and LID onset period (gray bar in I) for D2-MSN. [Kruskal-Wallis, ns]. Day 1: n=17, N=10; Day 4: n=29, N=10; Week 4: n=47, N=14. Data shown as mean ± SEM (B, C, F, G, I, J).

GCaMP fiber photometry allows measurement of cell-type specific neural activity in a stable population of neurons over the course of many sessions. However, it reflects population, rather than single-cell activity. Changes in bulk D1- and D2-MSN GCaMP signal after repetitive levodopa may result from alterations in ensemble size, amplitude of single-cell responses, or both. To distinguish these possibilities, we performed two new sets of experiments, assaying single-cell activity with the immediate early gene, c-Fos, and with single-unit electrophysiology. Administration of levodopa has been shown to induce immediate early gene expression in the striatum^47,49–51^. Specifically, c-Fos is increased in a subpopulation of striatal neurons, composed primarily of D1-MSNs, which are causally linked to LID^12,13^. To determine if a larger ensemble of D1-MSNs is recruited over the course of treatment, we sacrificed 6-OHDA-treated Drd1-tdTomato mice 2 hours after levodopa injection on day 1, day 4 and week 4 of treatment, and immunostained for c-Fos (Figure 3A). Consistent with previous studies^12^, the majority of c-Fos+ cells were D1-MSNs (62–86%), as measured by colocalization with tdTomato. We found that the density of c-Fos+ and colocalized c-Fos/tdTomato+ cells in the dorsolateral striatum did not differ between mice receiving varying durations of levodopa treatment (Figure 3B–C). These results suggest that a similar number of D1-MSNs are recruited by each levodopa administration, regardless of treatment duration.

To determine if single-cell firing responses to levodopa change over repetitive administration, we used *in vivo* single-unit electrophysiology in freely-moving parkinsonian mice. 6-OHDA-treated mice were implanted with an electrophysiology recording device in the DLS (Figure 3D). We performed single-unit recordings during levodopa treatment sessions on day 1, days 3–5 and week 4 of levodopa treatment. We identified and excluded putative interneurons based on spike waveform^10,52–54^. In line with previous findings^10^, we observed a variety of responses to levodopa across striatal neurons, including increased firing, decreased firing and no response. Based on previous optically labeled recordings in the same mouse model^10^, units that increased or decreased their firing in response to levodopa were classified as putative D1- or D2-MSNs, respectively (see Methods). No response units, which represented 4.2–12.5% of all units, were not analyzed further. As the onset phase showed greater differences over time, in terms of dyskinesia severity and striatal GCaMP signal, we focused on this phase in our analysis of single-unit firing responses. As the number of levodopa-activated D1-MSNs appeared to be stable across 4 weeks of treatment, we hypothesized that the levodopa-evoked firing of individual neurons would increase, producing the potentiation of D1 GCaMP responses across treatment. Indeed, putative D1-MSNs showed greater levodopa-evoked increases in firing at the single-unit level (Figure 3E–G). We also measured single-unit activity in putative D2-MSNs over repetitive levodopa administration (Figure 3H–I), but did not find significant changes in firing rate during LID onset (Figure 3J). These results suggest that alterations in D1-MSN responses following repetitive levodopa administration are primarily associated with changes in the amplitude of single-cell responses rather than alterations in ensemble size. While there are methodological and analytical limitations in the quantification of D2-MSN activity suppression or ensemble size, our data suggest that the evolution of D2-MSN responses seen with fiber photometry cannot be explained solely by enhanced suppression of individual neuron firing over the course of treatment.

**Figure 4.**
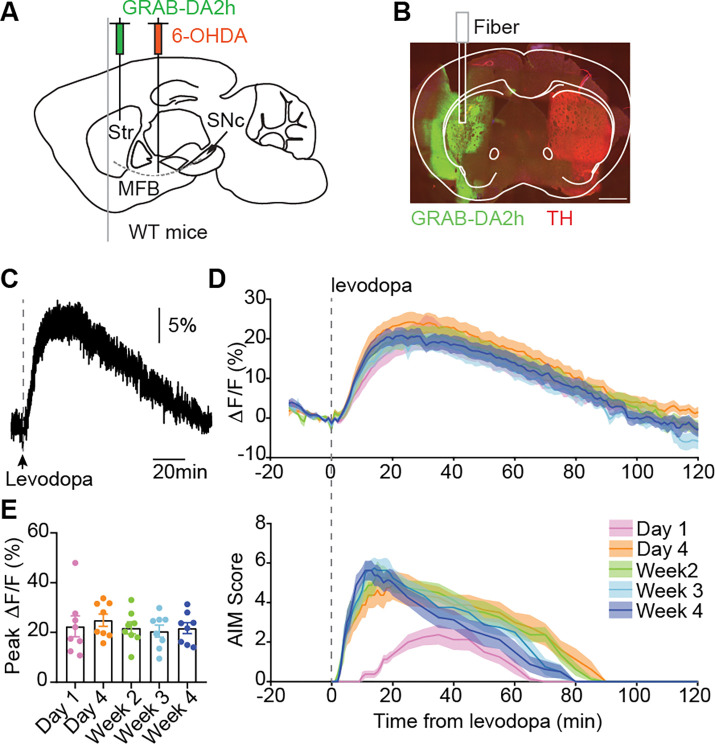
Repetitive levodopa does not alter striatal dopamine release. **(A)** Cartoon showing injection of AAV encoding GRAB-DA2h and fiber implant in the dorsolateral striatum, and injection of 6-OHDA into the medial forebrain bundle (MFB) of WT mice. **(B)** Representative coronal section showing GRAB-DA2h expression and TH loss. **(C)** Representative GRAB-DA2h signal in response to levodopa injection, as measured with fiber photometry. **(D)** Average normalized GRAB-DA2h (ΔF/F) responses to levodopa across treatment timepoints. **(E)** Peak normalized GRAB-DA2h ΔF/F responses to levodopa across timepoints. [RM one-way ANOVA, ns]. N=8 mice. Each dot is a session of one mouse.

We next sought to investigate the cellular mechanisms driving changes in D1- and D2-MSN responses to levodopa over repetitive treatment. Given the bidirectional levodopa responses of D1- and D2-MSNs, which potentiate in parallel between day 1 and day 4, one plausible mechanism is alterations in dopamine signaling. To determine whether levodopa-evoked dopamine release changes over this period, we performed fiber photometry with the fluorescent dopamine sensor GRAB-DA2h^55^. Wild type 6-OHDA-treated mice were injected with a virus encoding GRAB-DA2h and implanted with an optic fiber in the DLS (Figure 4A–B). We recorded dopamine release, as measured by GRAB-DA2h, in response to levodopa across four weeks of treatment. As expected, each levodopa administration increased dopamine release, with a time course that roughly matched the duration of increased movement and dyskinesia (Figure 4C). These responses were consistent with slow and sustained increases in dopamine, rather than with fast transient elevations. Most notably, the amplitude and timecourse of levodopa-evoked dopamine release was consistent over the course of four weeks of treatment (Figure 4D–E). These results suggest that changes in dopamine release are unlikely to explain the potentiation of MSN responses over the course of treatment.

### Repetitive levodopa administration increases excitatory transmission in D1-MSNs and reduces D2R sensitivity in D2-MSN

**Figure 5.**
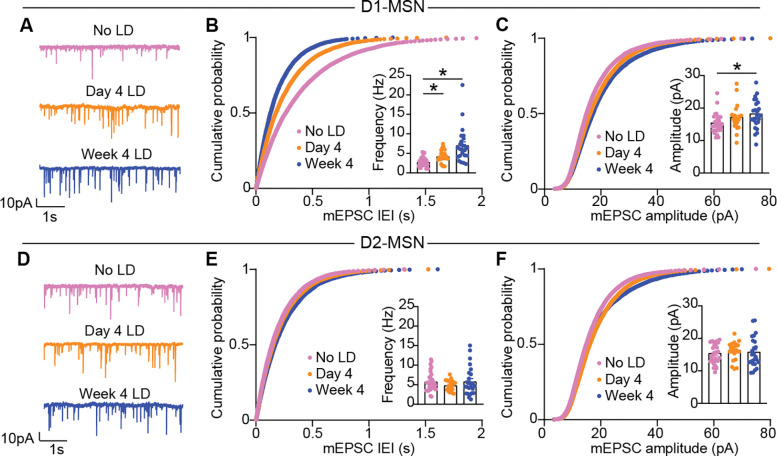
Repetitive levodopa treatments drive increased excitatory transmission onto D1-MSNs. 6-OHDA-treated animals were sacrificed at three different time points for *ex vivo* (slice) electrophysiology, either levodopa-naïve (“No LD”) or 24h after the last levodopa administration (Day 4 or Week 4). **(A)** Representative voltage clamp recordings of miniature excitatory postsynaptic currrents (mEPSC) in D1-MSNs across treatment timepoints. **(B)** Cumulative probability distribution of mEPSC Inter-Event-Intervals (IEI) in D1-MSNs. Inset: average mEPSC frequency. [Kruskal-Wallis, p<0.0001, post-hoc comparisons *p<0.05]. **(C)** Cumulative probability distribution of mEPSC amplitudes in D1-MSNs. Inset: average mEPSC amplitude. [Kruskal-Wallis, p=0.0158, post-hoc comparisons *p=0.0150]. No LD: n=30, N=11; Day 4 LD: n=22, N=6; Week 4 LD: n=24, N=6. **(D)** Voltage clamp recordings of mEPSC in D2-MSN across treatment timepoints. **(E)** Cumulative probability distribution of mEPSC Inter-Event-Intervals (IEI) in D2-MSN. Inset: average mEPSC frequency. [Kruskal-Wallis, ns]. **(F)** Cumulative probability distribution of mEPSC amplitudes in D2-MSN. Inset: average mEPSC amplitude. [Kruskal-Wallis, ns]. No LD: n=32, N=10; Day 4 LD: n=20, N=6; Week 4 LD: n=25, N=6. Data shown as mean for probability plots, and mean±SEM for insets.

Though levodopa-evoked dopamine release was consistent across treatment sessions, these repeated elevations may lead to changes in the properties of downstream circuit elements. One of the most well-known mechanisms through which dopamine can induce long-lasting alterations in striatal function is by controlling glutamatergic synaptic plasticity^36–38^. Transient increases in striatal dopamine in response to positive action outcomes drive the induction of corticostriatal synaptic plasticity^36,37,56^, thereby increasing the probability of repeating that action in the future^37,56^. In the dopamine-depleted striatum, the relationship between dopamine and action outcomes is disrupted, with exceptionally low striatal dopamine in the untreated state, and with levodopa treatment, dopamine increases for minutes to hours (see Figure 4). These alterations drive aberrant plasticity at striatal glutamatergic synapses after weeks of levodopa treatment^15,45^. However, it is unclear how striatal glutamatergic synapses are shaped over the course of levodopa treatment and how they relate to the progression of LID. To fill this gap, we performed *ex vivo* slice recordings in Drd1-tdTomato or D2-GFP 6-OHDA-treated mice, to allow targeting of D1- and D2-MSNs. We performed recordings in three treatment groups: treatment-naïve animals (mimicking baseline conditions on day 1 of levodopa), animals treated with levodopa for 3 days or 4 weeks (mimicking day 4 and week 4). Brain slices were prepared 24 hours after the last dose of levodopa, corresponding to the “OFF” levodopa state. This timing avoids acute effects of dopamine receptor activation during the “ON” state, thereby isolating chronic effects of repetitive levodopa exposure. To assess glutamatergic synaptic transmission onto D1- and D2-MSNs, we measured miniature excitatory postsynaptic currents (mEPSCs) in the presence of tetrodotoxin and picrotoxin (Figure 5A). Compared to the levodopa-naïve condition, we found an increase in the frequency of mEPSCs in D1-MSNs at both day 4 and week 4 of levodopa treatment (Figure 5B). There were small changes in mEPSC amplitudes, only evident between levodopa-naïve and week 4 groups (Figure 5C). In contrast, mEPSC frequency and amplitude in D2-MSNs were unchanged after repetitive levodopa (Figure 5D–F). As increased D1-MSN mEPSC frequency paralleled the potentiation in D1-MSN responses to levodopa and LID severity from day 1 to day 4, alterations in glutamatergic synaptic connectivity onto D1-MSNs are a strong candidate mechanism.

**Figure 6.**
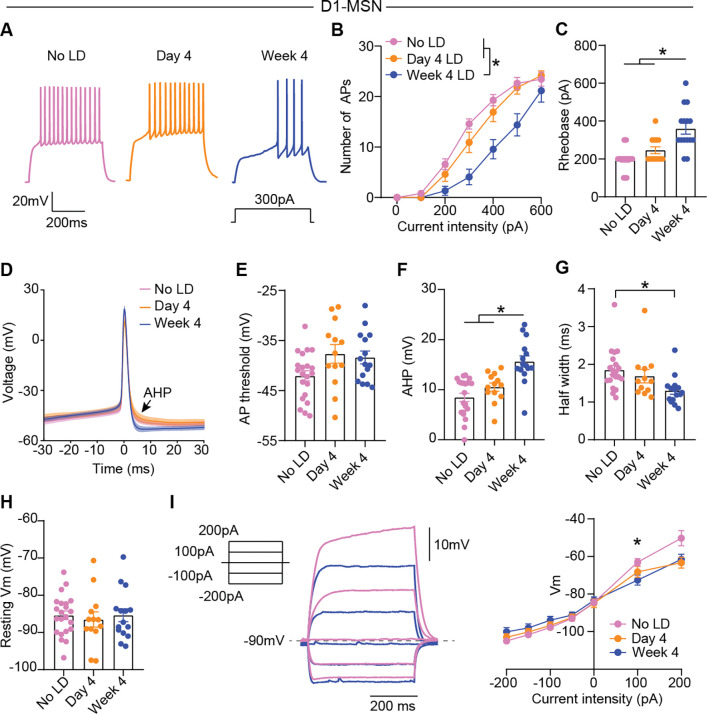
D1-MSN intrinsic excitability is reduced after repetitive levodopa treatment. 6-OHDA-treated animals were sacrificed at three different time points for *ex vivo* (slice) electrophysiology, either levodopa-naïve (“No LD”) or 24h after the last levodopa administration (Day 4 or Week 4). **(A)** Voltage responses to current injection in D1-MSN after different levodopa treatment duration. **(B)** Number of action potentials (APs) in response to depolarizing current steps. [Two-way ANOVA, treatment p=0.0001, interaction p<0.0001, post-hoc for 300, 400 and 500pA *p<0.05]. **(C)** D1-MSN rheobase across treatment duration. [Kruskal-Wallis, p<0.0001, post-hoc *p<0.05]. **(D)** Average action potential shape. **(E-H)** Action potential threshold (E), afterhyperpolarization (AHP, F), half width (G) and resting membrane potential (H). [Kruskal-Wallis, ns (E, H), p<0.0001 (F), p=0.0021 (G), post-hoc: *p<0.05]. **(I)** Voltage deflection to current injections. [Two-way ANOVA, interaction: p<0.0001, post-hoc between No LD and Week 4 at 100pA: *p=0.0211]. No LD: n=20–22, N=9; Day 4: n=12–13, N=6; Week 4: n=15, N=6. Data shown as mean±SEM. Each dot represents one cell.

Aside from controlling synaptic plasticity, dopamine can also influence D1- and D2-MSN activity by modulating cellular excitability through regulation of multiple ion channels^34,57,58^. Previous studies have shown that dopamine neuron degeneration and subsequent levodopa therapy can induce chronic alterations in MSN excitability^14,42^. However, precisely how MSN excitability is shaped over the course of levodopa treatment, particularly at key stages of LID progression, remains unclear. To directly test whether D1- and/or D2-MSN intrinsic excitability is altered after four days or four weeks of levodopa treatment, we again made *ex vivo* whole-cell patch clamp recordings from identified D1- or D2-MSNs in 6-OHDA-treated mice. In current-clamp recordings, we gave a series of current injections to evoke spiking (Figure 6A), and plotted the number of action potentials as a function of input current, as well as the minimum current needed to evoke spiking (rheobase). We found that D1-MSN excitability was unchanged after four days of levodopa administration, when the severity of LID has already peaked (Figure 6B–C). However, we found a marked reduction in excitability after four weeks of treatment, manifested as a reduced number of action potentials in response to depolarizing current steps and an increase in rheobase current (Figure 6A–C). This reduction in excitability is consistent with homeostatic adaptation to the elevated activity of D1-MSNs during levodopa treatment. In contrast, D2-MSNs did not show alterations in intrinsic excitability or basic membrane properties after four days or four weeks of treatment (Figure S4). These results indicate that D1-MSNs decrease their intrinsic excitability as a homeostatic mechanism in response repetitive levodopa treatment.

We next sought to identify candidate ionic conductances mediating the reduction in D1-MSN excitability after 4 weeks of treatment. Lower intrinsic excitability often correlates with increased potassium conductances, including those associated with spike-triggered calcium-activated potassium channels that mediate the afterhyperpolarization (AHP)^59–61^. We analyzed the action potential shape and passive membrane properties (Figure 6D–I). While there were no changes in action potential threshold (Figure 6E), we found that AHP amplitude was increased after four weeks of treatment, accompanied by a reduction in the spike half width (Figure 6F–G). D1-MSN resting membrane potential is mainly determined by constitutively active inwardly rectifying potassium channels (Kir2)^62^, which are modulated by D1 receptor activation^63,64^. However, we found no changes in resting membrane potential or voltage deflections in response to hyperpolarizing current steps that drove the membrane potential to more negative voltages where Kir2 is predominant (Figure 6H–I). Other channels are proposed as targets of dopaminergic modulation in D1-MSN, including voltage-dependent Kv1.2-containing channels that mediate the slowly-inactivating A-type potassium current, I_A_^58^. I_A_ rapidly activates in response to depolarizations, limiting D1-MSN transitions from down to up states^65^. An increase in I_A_ should therefore limit depolarizations in response to current injections. Indeed, subthreshold voltage deflections in response to depolarizing current steps were attenuated after four weeks of levodopa treatment (Figure 6I), consistent with possible upregulation of I_A_^34^. These results indicate that upregulation of I_A_ and calcium-activated potassium currents that mediate the AHP may underlie decreased D1-MSN excitability over levodopa treatment.

**Figure 7.**
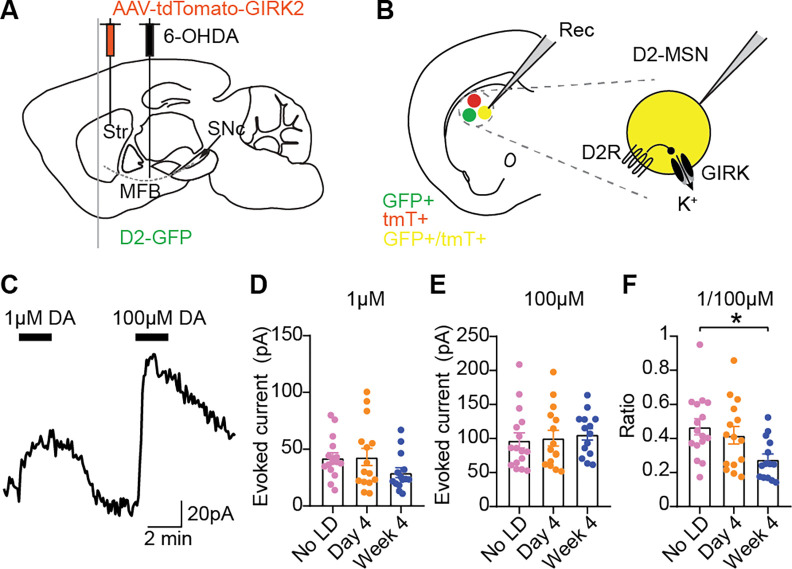
D2R sensitivity is reduced after 4 weeks of levodopa. D2-GFP mice were injected with an AAV encoding tdTomato-GIRK2 in the dorsolateral striatum, and injected with 6-OHDA in the MFB. **(A)** Experimental approach. **(B)** Schematic showing whole-cell recording of D2-MSNs expressing GIRK (GFP+/tdTomato+ cells). **(C)** Representative current trace showing responses to bath application of 1μM and 100μM dopamine (DA). **(D-F)** GIRK-mediated currents evoked by 1μM (D) or 100μM (E) dopamine and their ratio (F) in D2-MSN after different levodopa treatment timepoints. [F: Kruskal-Wallis, p=0.01, post-hoc: *p<0.05]. No LD: n=16, N=7; day 4: n=15, N=7; week 4: n=14, N=8. Data shown as mean±SEM. Each dot represents one cell.

While we identified a candidate cellular mechanism underlying the potentiation of D1-MSN responses to levodopa, we did not observe significant changes in the intrinsic or synaptic properties of D2-MSNs that might explain the potentiation of D2-MSN responses from day 1 to day 4, and the progressively faster recovery from day 4 to week 4 (see Figure 2). One possibility is that dopamine signaling through the D2R is altered over repetitive exposures to levodopa. To directly test this hypothesis, we overexpressed an effector of D2R signaling in many cell types, the G-protein-activated inwardly rectifying potassium channel (GIRK2)^66,67^. We injected AAV encoding GIRK2-tdTomato in the DLS of 6-OHDA-treated D2-GFP mice (Figure 7A). Again, mice were either left untreated (no LD), or treated with levodopa for 3 days or 4 weeks. We then performed whole-cell voltage-clamp recordings (Vh=−60mV) of GFP+/tdTomato+ cells 24 hours after the last dose of levodopa, in the presence of blockers of GABA, glutamate, D1 and muscarinic receptors to isolate the effect of dopamine on D2R^66^ (Figure 7B). Bath application of dopamine induced outward currents through D2R coupling to GIRK channels (Figure 7C). We applied dopamine at 1μM (near the EC50 of levodopa-naive 6-OHDA-treated mice) followed by a saturating concentration of 100μM to account for intercellular variability in GIRK expression (Figure 7C–E). We found that the sensitivity of D2R is not increased after four days of levodopa treatment, as evidenced by a stable ratio of GIRK currents evoked by 1μM dopamine relative to 100μM (Figure 7F). However, after 4 weeks, a reduced ratio of dopamine-evoked GIRK currents indicated a decrease in D2R sensitivity (Figure 7F). These results suggest that a decrease in D2R sensitivity may contribute to the faster recovery from inhibition of D2-MSNs and the more rapid resolution of AIMs after four weeks of treatment.

## DISCUSSION

In this study, we combined behavior with *in vivo* and *ex vivo* techniques to understand the mechanisms associated with the progression of LID over the course of a month of levodopa therapy. We found that repetitive levodopa administration drives two major changes in behavior: (1) it worsens the severity and sharpens the onset of LID, and (2) accelerates the resolution of LID. Moreover, we revealed that these alterations are mediated by distinct long-lasting alterations in striatal function: (1) the more rapid onset and severity of LID is associated with a potentiation of D1-MSN and D2-MSN responses to levodopa and with enhanced glutamatergic synaptic inputs onto D1-MSNs; (2) the more rapid LID offset is linked to a faster recovery of D2-MSN activity and reduced D2R sensitivity.

People with PD gradually develop LID, which then worsens over years of levodopa treatment. Despite its clinical significance, the mechanisms driving this worsening remain largely unaddressed. Clinical studies suggest that advancing neurodegeneration, treatment duration and levodopa dosage are key risk factors for the development and worsening of LID^7,9^. However, disentangling their individual contributions remain challenging, as these factors evolve simultaneously in patients. By utilizing the 6-OHDA mouse model of PD, we were able to isolate the specific effects of repetitive levodopa treatment, as the extent of neurodegeneration and dosage remain fixed in this model. Our results demonstrate that repetitive levodopa worsens LID, resulting in more rapid onset and severe dyskinesia with each dose. Based on multiple prior studies suggesting that the activity of D1-MSNs is causally related to LID^12,21,68^, we hypothesized this increase in LID severity might be mediated by a concomitant increase in levodopa-evoked D1-MSN activity. Indeed, we found that levodopa-evoked increases in D1-MSN activity enhance over treatment, using both GCaMP fiber photometry and single-unit electrophysiology. These two approaches served complementary purposes in our study, indicating that not only does the ensemble neural activity increase over the course of treatment, but the levodopa-evoked firing rates of individual D1-MSNs increase over treatment. Though c-Fos immunohistochemistry may not fully capture the ensemble of D1-MSNs activated by levodopa, we did not see a change in the number of c-Fos-labeled D1-MSNs in dyskinesia over the course of treatment. Our findings suggest that potentiation of D1-MSN firing rate responses to levodopa could contribute to the worsening of LID over time.

The potentiation of D1-MSN responses that we observed *in vivo* could be caused by an increase in striatal dopamine signaling, intrinsic excitability, and/or synaptic plasticity. Our results using GRAB-DA2h fiber photometry show that alterations in striatal dopamine release are not a likely mechanism. One limitation of this approach is the sensitivity of optical dopamine sensors. We selected GRAB-DA2h due to its high affinity for dopamine^55^, which aligns well with the striatal extracellular dopamine concentrations measured via microdialysis in 6-OHDA-treated rats following administration of a similar dose of levodopa^69^. However, GRAB-DA fiber photometry may not be able to resolve small differences in dopamine release, or account for spatial heterogeneity. Alternative techniques, such as fast-scan cyclic voltammetry^70^, multi-fiber photometry^71^, or 2-photon imaging across a field of view^72^ might better capture changes in the dynamics of dopamine release. As dopamine increases the excitability of D1-MSNs via the D1 receptor^16,34,73^, the increase in D1-MSN activity may be mediated by enhanced postsynaptic responses.

An increase in the intrinsic excitability of D1-MSNs over the course of treatment could amplify excitatory inputs and explain higher levodopa-evoked firing rates. However, we did not see an increase in the excitability of D1-MSNs over time. Instead, we found that excitatory synaptic input onto D1-MSNs was increased over the course of treatment. Previous studies have measured striatal synaptic transmission in parkinsonian and levodopa-treated animals, finding evidence for increased synaptic strength in animals with LID^15,45^, but these studies did not take into account the effect of time as we did in our study. Repeated and prolonged elevation of striatal dopamine may hijack existing mechanisms for striatal synaptic plasticity, promoting aberrant potentiation of excitatory synapses onto D1-MSNs^15,45^. Dopamine-dependent potentiation of corticostriatal synapses onto D1-MSNs plays a crucial role in healthy motor learning, reinforcing actions associated with positive outcomes^37,40^. Consequently, corticostriatal synapses onto D1-MSNs encoding rewarded actions are strengthened. However, this mechanism becomes dysregulated in LID, uncoupling dopamine signaling from natural action-outcome associations. As a result, rather than selectively enhancing plasticity in action-relevant D1-MSNs, sustained dopamine elevations may instead promote aberrant plasticity in a subset of D1-MSNs that are intrinsically more vulnerable. Heightened vulnerability may stem from intrinsic properties, such as higher D1 receptor expression relative to neighboring D1-MSNs^13^ or from synaptic properties, such as enhanced corticostriatal inputs. Either of these factors may also correlate with the recruitment of these vulnerable D1-MSNs during episodes of dyskinesia.

Although D1-MSNs play a crucial role in LID, studies have also highlighted a role for D2-MSNs^16,17,20,30^. In parallel with the augmentation of D1-MSN responses, we found a reciprocal change in D2-MSN activity: stronger suppression of activity by levodopa over time. Importantly, we observed this change using GCaMP fiber photometry, but not in single-unit electrophysiological recordings. While fiber photometry enables longitudinal monitoring of neuronal activity in the same ensemble of cells, it may not accurately reflect changes in somatic firing^74^. Moreover, low-firing neurons—detectable via electrophysiology—may contribute less to the overall GCaMP signal. Despite the limitations of both approaches in detecting activity suppression, our findings suggest that stronger D2-MSN suppression may not be explained solely by changes in firing rate. Other contributing factors may include an increase in the number of suppressed cells or alterations in non-somatic calcium dynamics.

This stronger suppression of D2-MSN activity closely correlated with LID severity during the onset phase. As with the changes in D1-MSNs, this alteration in D2-MSNs could be mediated by dopamine signaling, intrinsic or synaptic properties. Strikingly, we did not find alterations in D2 receptor sensitivity, intrinsic excitability, or excitatory synaptic transmission during this early phase of treatment. An alternative mechanism would be increased synaptic inhibition of D2-MSNs. There are several sources of inhibitory synaptic input to D2-MSNs^75–77^, some of which are altered in parkinsonian mice^11,43,77,78^. However, the mirrored responses in D1- and D2-MSNs make direct lateral inhibitory connections between D1- and D2-MSNs a strong candidate. Even without an increase in the connection rate or strength of these synapses, the increasing levodopa-evoked activity of D1-MSNs could drive the increased levodopa-evoked inhibition of D2-MSNs over the course of treatment. In turn, decreased D2-MSN activity could lead to disinhibition of D1-MSNs via lateral connections. In line with this idea, recent studies indicate that the strength of these lateral inhibitory connections is shaped by both dopamine neuron loss and levodopa treatment^11,77^, with their dysregulation emerging as a key driver of LID^11^. Finally, cholinergic interneurons may also play a key role in this process, as their activity can regulate D1- and D2-MSNs^15,62,79–82^.

In addition to observing a more rapid and severe onset phase of LID, we saw that the offset of LID hastened between day 4 and week 4 of treatment. It is possible that this change relates to the development of wearing off and “ON-OFF” fluctuations in PD, which are defined as a shorter duration of levodopa action and rapid transitions from an “ON” state (relatively normal movement) to “OFF” state (marked parkinsonism, including freezing), respectively. These motor fluctuations arise over the course of levodopa treatment in PD, with similar risk factors to LID^83^. Interestingly, we found that the more rapid offset of LID is not linked to changes in D1-MSN activity but rather to kinetics of D2-MSN activity, which recovers from inhibition more rapidly after repetitive levodopa treatment. Paralleling this change, we found that the sensitivity of D2 receptors is reduced in D2-MSN after 4 weeks of treatment, which may contribute to the more rapid recovery. Other mechanisms could also contribute to the changes in D2-MSN activity during the offset period, including changes in ligand-dependent D2 receptor internalization^84^ and state-dependent changes in glutamatergic transmission^16^.

Some changes in the intrinsic and synaptic properties of MSNs can explain the worsening of LID over the course of levodopa treatment. However, other changes may be homeostatic in nature. One such change that we observed was a reduction in the intrinsic excitability of D1-MSNs, consistent with a previous report^14^. The decrease in D1-MSN excitability developed between day 4 and week 4 of levodopa treatment, and was associated with a larger afterhyperpolarization (AHP) and a reduction in spike half-width. These findings are consistent with increased big conductance (BK) and/or small conductance (SK) calcium-activated potassium currents, which may account for the reduced spike count once the threshold is reached. Additionally, our analysis using subthreshold current injections suggests that upregulation of A-type potassium currents may contribute. Interestingly, recent research has shown that BK, SK, and I_A_ currents are regulated by endogenous dopamine via D1 receptor stimulation^34^. An increased availability of these currents may render D1-MSNs more susceptible to dopamine modulation during the “ON” state. Thus, while the increase in these currents may be a homeostatic reaction to sustained elevations in dopamine signaling, it may be maladaptive. Indeed, despite the reduction in D1-MSN intrinsic excitability after 4 weeks of treatment, peak levodopa-evoked firing rates and GCaMP signals were similar in D1-MSNs at day 4 and week 4.

Prevailing models of basal ganglia function propose that the co-activation of D1-MSNs and D2-MSNs is essential for action selection, with D1-MSNs facilitating desired motor programs and D2-MSNs suppressing competing ones^25^. A prediction of this model is that achieving antiparkinsonian effects without dyskinesia requires restoring the balanced co-activation of D1- and D2-MSNs. Our findings support this prediction. Following the first dose of levodopa (day 1), before the onset of LID, we observed a brief period –lasting about 10 minutes– where levodopa produces antiparkinsonian effects (as shown by velocity increases) without dyskinesia (see Figure 1B and Figure S1A). This phase is characterized by an increase in the activity of D1-MSNs, but also a transient increase in the activity of D2-MSNs (see day 1 trace on Figure 2K). As dyskinesia emerges, D1-MSN activity remains elevated, while D2-MSN activity is markedly suppressed (see Figure 2K). This suppression may be crucial for the transition from antiparkinsonian effects to LID, by disrupting coactivation, and thus impairing the ability of D2-MSNs to inhibit competing movements. A similar pattern is observed during the offset phase: while D1-MSN activity remains high over treatment, the key physiological correlate of LID resolution (and its concomitant increase in locomotor velocity) appears to be recovery of D2-MSN activity. Taken together, these observations suggest that while D1-MSN activation is necessary for both the therapeutic effects of levodopa and the expression of dyskinesia, the behavioral outcome at comparable levels of D1-MSN activity appears to be shaped by D2-MSNs: their suppression promotes LID, while their co-activation supports therapeutic locomotion. Overall, these findings reinforce the idea that both striatal populations play essential roles in LID pathophysiology and that excitatory and inhibitory synaptic connections contribute to the progression of LID over the course of levodopa treatment in PD.

## STAR METHODS

### RESOUCE AVAILABILITY

#### Lead contact

Requests for additional information, resources, or reagents should be directed to and will be fulfilled by the Lead contact, Alexandra Nelson (alexandra.nelson@ucsf.edu).

#### Materials availability

This study did not generate any new unique materials.

#### Data and code availability

The data, code, protocols, and key lab materials used and generated in this study are listed in a Key Resource Table alongside their persistent identifiers. All data and code generated from this publication are available on Zenodo (DOI: 10.5281/zenodo.15446526). Any additional information required to reanalyze the data reported in this paper is available from the lead contact upon request.

### EXPERIMENTAL MODEL AND STUDY PARTICIPANT DETAILS

#### Animals

All animal procedures were approved by the University of California San Francisco Institutional Animal Care and Use Committee (IACUC). Animals were housed under a 12h light/dark cycle with access to food and water ad libitum. For all experiments, both male and female C57Bl/6J mice, aged 3–12 months, were used. For *ex vivo* electrophysiology experiments and c-Fos staining, D1-tdTomato or D2-GFP mice were used. The following mouse lines were used for *in vivo* experiments: GCaMP fiber photometry: D1-Cre and A2a-Cre; GRAB-DA fiber photometry: wild type; *in vivo* single-unit electrophysiology: D1-Cre, A2a-Cre and wild type. Age-matched littermates were randomly assigned to experimental and control groups.

### METHOD DETAILS

#### Surgical Procedures

A detailed surgical protocol can be found at (DOI: dx.doi.org/10.17504/protocols.io.dm6gpdw1dgzp/v1). Briefly, three- to six-month old mice were anesthetized with a combination of ketamine/xylazine (40/10 mg/kg IP) and inhaled isoflurane (1%). After placement in the stereotaxic frame (Kopf Instruments), the scalp was opened, and a mounted drill was used to create a hole over the left medial forebrain bundle (MFB). Using a 33-gauge needle (WPI) or a syringe with removable glass needle (Hamilton) and Micro4 pump (WPI), 1 μL of 6-hydroxydopamine (6-OHDA, Sigma-Aldrich, 5 μg/μl in normal saline) was injected unilaterally into the MFB (−1.0 AP, −1.0 ML, −4.9 DV) at a rate of 0.2 μl/min. To reduce toxicity to other monoaminergic axons, desipramine (Sigma-Aldrich, 25mg/kg IP) was administered 30 min prior to 6-OHDA injection. After the surgery, animals received daily saline injections and were provided high-fat dietary supplements (Diet-Gel, peanut butter, forage mix) for a 1–2 week recovery period.

Viral injections were performed during the same surgical procedure as the MFB injection described above. A mounted drill was used to create a hole over the left dorsolateral striatum (DLS, +0.8 AP, −2.4 ML, −3 DV). For *ex vivo* electrophysiology experiments with GIRK overexpression, D2-GFP mice were injected with 500 nL AAV9-hsyn-TdTomato-T2A-mGIRK2–1-A22A-WPRE-bGH (undiluted, UPenn Vector Core) into the DLS. For fiber photometry experiments, D1-Cre or A2A-Cre mice were injected with 500 nL of a Cre-dependent GCaMP6s (diluted 1:9, UPenn Vector Core) into the DLS, whereas wild type mice were injected with 500 nL of AAV9-hsyn-GRAB-DA2h (undiluted, Addgene 140554) and implanted with a fiber-optic ferrule (0.4 mm, Doric Lenses) above the DLS (−2.7 DV). Fibers were secured with dental cement (Metabond) and dental acrylic (Ortho-Jet or Henry Schein).

For *in vivo* single-unit electrophysiology experiments, the scalp was reopened 3–4 weeks after the initial surgeries to implant the recording device. Two different recording devices were used: either a fixed 32-channel electrode array (Innovative Neurophysiology) or a high density 64-channel silicon probe (Cambridge Neurotech). For 32-channel electrode array implantation, three additional holes were drilled for two skull screws (FST) and a ground wire. The array was slowly inserted through the craniectomy into the DLS (−2.5 DV). A thin layer of dental cement (Metabond) was applied to the surface of the skull and dental acrylic (Ortho-Jet or Henry Schein) was applied to cover all exposed hardware. For silicon probe implantation, a movable 2-shank 64-channel high-density silicon probe (model H6, Cambridge Neurotech), was mounted on a movable microdrive and implanted above the DLS, and a ground wire above the cerebellum. A head bar was also attached to the skull to allow tethering without damaging the probe. The recording electrodes were stained with DiI or DiO lipophilic dyes (Thermo Fisher) prior to implantation for post hoc identification of the electrode track. The experiments were performed at least one day after implantation. For some timepoints (like day 3–5 and week 4), where multiple days were recorded, the probe depth was finely adjusted by moving the microdrive so that different neurons were recorded each day.

#### Behavior

Details of the behavioral assessments can be found at (DOI: https://www.protocols.io/view/behavioral-testing-open-field-and-dyskinesia-scori-6qpvr67oovmk/v1). Briefly, after a 6 weeks post-surgical baseline period, animals began daily treatment. Animals were placed on an open field arena (25 cm diameter cylinder) and video recorded with a side and a top camera. Gross movement was quantified using video-tracking software (Noldus Ethovision). Parkinsonian animals receiving levodopa developed robust levodopa-induced dyskinesia (LID). Dyskinesia was quantified by the experimenter using the Abnormal Involuntary Movement score (AIMs), a standardized metric that considers dyskinesia across axial, limb, and orolingual body segments. As described by Cenci and Lunblad (2007), dyskinesia severity in each body segment was quantified during 60 s observation windows as follows: 0 = normal movement, 1 = abnormal movement for < 50% of the time, 2 = abnormal movement for > 50% of the time, 3 = abnormal movement for 100% of the time, but can be interrupted, and 4 = continuous, uninterruptable abnormal movement. The total AIM score represents the summation of the AIM score for each body segment (axial, limb, orolingual), for a maximum possible score of 12. For *ex vivo* experiment animals, AIMs were scored every 20 minutes for a 2-hour period. For *in vivo* experiments (GCaMP & GRAB-DA fiber photometry and single-unit electrophysiology), AIMs were scored every minute for the first 20 minutes after injection, and every 5 minutes thereafter to complete 120 minutes.

#### Pharmacology

Details for the preparation of pharmacological agents can be found at (DOI: dx.doi.org/10.17504/protocols.io.e6nvw1nbzlmk/v2). For all the experiments, 5 mg/kg levodopa (Sigma-Aldrich) was co-administered with 2.5 mg/kg benserazide (Sigma-Aldrich) and prepared in normal saline. Levodopa was given via IP injection 5 days per week. For *in vivo* experiments, daily levodopa injections continued for 4 weeks. For *ex vivo* experiments, levodopa was administered either for 3 days or for 4 weeks before the experiment. Picrotoxin (Sigma-Aldrich) was dissolved in warm water at 5 mM and added to ACSF for a final concentration of 50 μM. SCH23390 (Tocris) was dissolved in water at 10 mM and added to ACSF for a final concentration of 10 μM. AP5 (Abcam) was dissolved in water at 50 mM and added to ACSF for final concentrations of 50 μM. Scopolamine (Tocris) was dissolved in water at 200 μM and added to ACSF for final concentrations of 200 nM. CGP55845 (Tocris) was dissolved in water at 300 μM and added to ACSF for final concentrations of 300 nM. Dopamine (Sigma-Aldrich) was dissolved in water with 0.1% ascorbic acid at 1 or 100 mM and added to ACSF for a final concentration of 1 or 100 μM. CNQX (Sigma-Aldrich or Tocris) was dissolved in water at 10 mM and added to ACSF for a final concentration of 10 μM. TTX (Tocris) was dissolved in water at 1 mM and added to ACSF for a final concentration of 1 μM. Sulpiride (Tocris) was dissolved in water at 10 mM and added to ACSF for a final concentration of 10 μM.

#### Fiber photometry

A detailed protocol for fiber photometry can be found at https://doi.org/10.17504/protocols.io.8epv59dbjg1b/v1. Animals were habituated to IP injections and tethering for at least 30 minutes/ session for 2 sessions before starting the experiments. Mice were placed in a circular open field (25 cm diameter) and recorded for a baseline period of 30 minutes before saline or levodopa injection. For saline sessions, GCaMP signals were recorded for 1 hour after injection. For levodopa sessions, GCaMP signals were recorded for 2 hours after injection. A side camera and a top camera (20 fps, Imaging Source) allowed for post-hoc quantification of gross movement using an offline video-tracking software (Noldus Ethovision). Fine behavior was manually scored by the experimenter (AIM score). Fiber photometry signals were acquired through implanted 400 μm optical fibers, using an LED driver system (Doric). Following signal modulation, 405 nm (isosbestic) and 465 nm signals were demodulated via a lock-in amplifier (RZ5P, TDT), visualized, and recorded (Synapse, TDT). Videos were synchronized with simultaneous photometry recordings via TTL pulses triggered by the video tracking software. IP injection times were synchronized with simultaneous photometry recordings via manually triggered TTL pulses.

#### In vivo electrophysiology

*In vivo* extracellular recordings were performed using two different recording devices: a fixed 32-channel microwire (35 mm tungsten) array (Innovative Neurophysiology) or a drivable high-density silicon probe (Cambridge Neurotech chronic 64 channel H6, 2 shanks, 9 mm length). A detailed protocol for in vivo electrophysiology can be found at: dx.doi.org/10.17504/protocols.io.36wgq641ylk5/v1. Briefly, mice were habituated to the open field (25 cm diameter), tethering and IP injections (saline). The animal’s gross behavior was recorded by video tracking software (Noldus Ethovision). Fine behavior was manually scored by the experimenter (AIM score). Recording sessions consisted of a 30-minute baseline period, followed by IP injection of levodopa. Electrophysiological recordings continued for at least 80 minutes post-injection. Behavioral measurements were synchronized with simultaneous electrophysiological recordings via TTL pulses triggered by the video tracking software and recorded by the electrophysiology system.

For recordings using fixed a 32-channel microwire array the animal was placed in the open-field while tethered via a lightweight, multiplexed headstage cable (Triangle Biosystems) attached to a low-torque electrical commutator (Dragonfly) to allow free movement. Single unit activity from microwires was recorded using a 32-channel recording system (MAP system, Plexon). Spike waveforms were filtered at 154–8800 Hz and digitized at 40 kHz. The experimenter manually set a threshold for storage of electrical events. To verify the location of the optrode array in DLS, mice were deeply anestethized after the last recording session and, prior to transcardia perfusion, electrolytic lesions were made to mark electrode tips using a solid state, direct current Lesion Maker (Ugo Basile), by applying 100 μA for 5 s per microwire. Mice were then terminally anesthetized with ketamine/xylazine (200/40 mg/kg i.p.), transcardially perfused with 4% paraformaldyde (PFA), and the brain dissected from the skull. The brain was post-fixed overnight in 4% PFA and then placed in 30% sucrose at 4°C for histology processing. A subset of these recordings were reported previously, with a different scientific question and analysis approach^10,13^.

For high-density silicon probe recordings, we recorded the signals at 30 kHz using an INTAN system (RHD2000 USB Interface Board, INTAN Technologies). Animals were tethered via a lightweight ultra-thin SPI interface cable (INTAN Technologies) attached to an electrical commutator (Doric). Signals were digitized at 30 kHz. To verify the location of the probe tracks, mice were terminally anesthetized with ketamine/xylazine, perfused with 4% PFA, and the brain dissected for histology processing.

#### Ex vivo electrophysiology

A detailed protocol for *ex vivo* electrophysiology can be found at (DOI: dx.doi.org/10.17504/protocols.io.kqdg3k6n1v25/v1). To prepare acute brain slices, mice were anesthetized with ketamine/xylazine and transcardially perfused with 95%O_2_/5%CO_2_ oxygenated, ice-cold glycerol-based artificial cerebrospinal fluid (ACSF) containing (in mM): 250 glycerol, 2.5 KCl, 1.2 NaH_2_PO_4_, 10 HEPES, 21 NaHCO_3_, 5 D-Glucose, 2 MgCl_2_, 2 CaCl_2_. Following decapitation, brains were dissected and sequential coronal slices (275 μm) containing the striatum were collected with a vibratome (Leica). Slices were immediately transferred to a holding chamber containing warm (33°–34°C), carbogenated ACSF containing (in mM): 125 NaCl, 26 NaHCO_3_, 2.5 KCl, 1.25 NaH_2_PO_4_, 12.5 D-Glucose, 1 MgCl_2_, 2 CaCl_2_. Slices were incubated for 45 min and then held at room temperature (22°–24°C) until used for recording.

For all recordings, slices were transferred to a recording chamber superfused (~2 mL/min) with carbogenated ACSF (31°–33°C). Medium spiny neurons were targeted for recordings using differential interference contrast (DIC) optics on an Olympus BX 51 WIF microscope. In Drd1a-tdTomato mice, direct pathway neurons were identified by their tdTomato-positive somata. Conversely, indirect pathway neurons were identified by their medium-sized tdTomato-negative somata. In D2-GFP mice, indirect pathway neurons were identified by their GFP-positive somata and direct pathway neurons were identified by their GFP-negative, medium-sized somata. Fluorescent-negative neurons with interneuron-like physiological properties were excluded from the dataset.

Medium spiny neurons were patched in the whole-cell configuration using borosilicate glass electrodes (2–5 MΩ). For voltage-clamp measurements of miniature excitatory postsynaptic currents (mEPSC) at a holding potential of −70mV, electrodes were filled using a cesium-based internal solution containing (in mM): CsMeSO3, 15 CsCl, 8 NaCl, 0.5 EGTA, 10 HEPES, 2 MgATP, 0.3 NaGTP, 5 QX-314, pH 7.3. Picrotoxin (50 μM) and TTX (1 μM) was added to the external solution. For current-clamp experiments, electrodes were filled using a K-based internal solution containing (in mM): 130 KMeSO_3_, 10 NaCl, 2 MgCl_2_, 0.16 CaCl_2_, 0.5 EGTA, 10 HEPES, 2 MgATP, 0.3 NaGTP, pH 7.3). Picrotoxin (50 μM) was added to the external solution. After whole-cell break in, cells were given at least 5 min to dialyze internal solution before recording. Whole-cell recordings were made using a MultiClamp 700B amplifier (Molecular Devices) and digitized with an ITC-18 A/D board (HEKA). Data were acquired using Igor Pro 6.0 software (Wavemetrics) and custom acquisition routines (mafPC, courtesy of M.A. Xu-Friedman). For voltage-clamp measurements of dopamine-evoked GIRK currents, whole-cell recordings were made from double fluorescent (GFP^+^ and tdTomato^+^;GIRK2^+^) MSNs. Electrodes were filled using an internal solution containing (in mM): 135 mM D-Gluconic Acid (K), 2 mM MgCl_2_, 0.1 mM CaCl_2_, 10 mM HEPES (K), 10 mM BAPTA-tetrapotassium, 1 mg/ml ATP, 0.1 mg/ml GTP, and 1.5 mg/ml phosphocreatine (pH 7.3, 285 mOsm). GFP^+^/GIRK2^+^ MSNs were held at −60 mV. Picrotoxin (50 μM), SCH23390 (10 μM), AP5 (50 μM), Scopolamine (200 nM), CGP55845 (300 nM) and CNQX (10 μM) were always present in the external solution to isolate D2 receptor mediated increases in GIRK currents. Dopamine was applied at 1 μM by bath perfusion. After ~3–4 minutes dopamine was washed out with ACSF for 4–5 minutes and then 100 μM dopamine was applied.

#### Histology and microscopy

A detailed protocol for preparation of histological sections can be found at (DOI: dx.doi.org/10.17504/protocols.io.b9ubr6sn). After behavioral experiments, mice were anesthetized with ketamine/xylazine and transcardially perfused with 4% paraformaldehyde (PFA) in phosphate buffer solution (PBS). Following perfusion, brains were dissected and post-fixed overnight in 4% PFA, then transferred to 30% sucrose and stored at 4°C for cryoprotection. Brains were then sliced coronally into 30 μm sections on a freezing microtome (Leica).

After *ex vivo* electrophysiology experiments, 275 μm slices were stored overnight in 4% PFA. The following day, slices were transferred to 30% sucrose and sub-sectioned into 50 μm sections on a freezing microtome (Leica).

For immunohistochemistry, the tissue was blocked in 3% normal donkey serum (NDS) and permeabilized with 0.1% Triton X-100 on a room-temperature (RT) shaker. Primary anti-bodies (Rabbit anti-TH, Pel-Freez Biologicals, 1:1000); rabbit anti c-Fos (Cell Signaling Technology, 1:1000) were added to the NDS and tissue was incubated overnight on a 4°C shaker. Tissue was then incubated in secondary antibodies (Donkey anti-rabbit Alexa Fluor 488, 594 or 647, 1:500) overnight (16 h) on a 4°C shaker before being washed and mounted (Vectashield Mounting Medium) onto glass slides for imaging. Images (4x, 10x, or 40x) were obtained using a Nikon 6D conventional widefield microscope or an Olympus Fluoview FV3000 confocal microscope. In 6-OHDA treated mice, the extent of dopamine depletion was confirmed by TH immunohistochemistry. Adequate viral expression (GCaMP, GRAB-DA, GIRK-tdTomato) was confirmed.

### QUANTIFICATION AND STATISTICAL ANALYSIS

#### Statistics

Details can be found in Table S1. Statistical tests were performed using GraphPad Prism 10. All data are presented as the mean ± SEM, with “n” referring to the number of cells and “N” referring to the number of animals.

#### Behavior

Dyskinesia was quantified using the Abnormal Involuntary Movement score (AIMs), as described above. The motion of the mouse was tracked using an offline video tracker software (Noldus, Ethovision), that tracked the center of gravity of the animal in the open field arena. Mouse velocity was quantified as the average velocity in 1-minute bins (Figure S1). Changes in AIMs and velocity (Figure 1 and S1), were compared across treatment timepoints using repeated-measures one-way ANOVA, with a post-hoc Tukey test.

#### Fiber photometry

Offline, the 405 nm signal was fit to the 465 nm signal using a first-degree polynomial fit (Matlab) to extract the non-calcium-dependent signal (due to autofluorescence, fiber bending, etc.). The fitted 405 nm signal was then subtracted from the 465 nm signal to generate a motion-corrected signal. For GCaMP experiments, we corrected for the gradual bleaching observed over the ~2.5-hour recordings by fitting an exponential curve to the signal and normalizing it by the fit. The resulting signal was then smoothed to reduce noise. To detect GCaMP transients, we calculated the envelope of the ΔF/F signal to capture meaningful fluctuations, and identified peaks and valleys of the envelope. Transients were defined as valley-to-peak difference exceeding 2% ΔF/F. The baseline-subtracted transient count or ΔF/F values were binned in 1-minute intervals and averaged across animals (Figure 2; Figure S3). Changes in ΔF/F or ΔTransients (Figures 2 and S2) across treatment timepoints were compared using repeated-measures one-way ANOVA, with a post-hoc Tukey test. The linear correlation between ΔF/F or ΔTransients and AIMs was calculated using Pearson correlation (Figure 2 and S2). For GRAB-DA experiments, ΔF/F was extracted, binned into 1-minute intervals, and aligned to levodopa injection (Figure 4). Changes in peak ΔF/F (Figures 4) across treatment timepoints were compared using repeated-measures one-way ANOVA, with a post-hoc Tukey test.

#### In vivo electrophysiology

For recordings performed using 32-channel microwire arrays (Innovative Neurophysiology) and an associated 32-channel recording system (MAP system, Plexon), single units (SUs) were identified offline by manual sorting into clusters (Offline Sorter, Plexon). Waveform features used for separating units were typically a combination of valley amplitude, the first three principal components (PCs), and/or nonlinear energy. Clusters were classified as SUs if they fulfilled the following criteria: (1) < 1% of spikes occurred within the refractory period and (2) the cluster was statistically different (p < 0.05, MANOVA using the aforementioned features) from the multi- and other single-unit clusters on the same wire. For recordings performed with 64-channel high-density probes and INTAN system, we classified SUs by automated spike sorting (KiloSort, https://github.com/cortex-lab/Kilosort). SUs were then manually curated using Phy (https://github.com/cortex-lab/phy). SUs were then classified as putative medium spiny neurons (MSNs) as previously described^10,52–54^, using features of the spike waveform (peak to valley and peak width), as well as inter-spike interval distribution. Only putative MSNs were included in subsequent analyses.

Firing rate was averaged in 1-minute bins. Modulation of firing rate by levodopa was determined by comparing SU firing rates before and after levodopa administration. The 30-minute baseline period was compared to a 30-minute period following drug injection (10–40 minutes post-injection). Following levodopa administration, SUs were categorized into three broad groups as follows, based on significant changes in firing rate (p < 0.01, Wilcoxon rank-sum test (denoted Mann-Whitney)) following levodopa treatment: putative D1-MSNs (increase in firing rate), putative D2-MSNs (decrease in firing rate), or no change units (nonsignificant change in firing rate).

Changes in the levodopa-evoked increases or decreases in firing rates across treatment timepoints were compared using a Kruskal-Wallis test with a Dunn’s post-hoc (Figure 3).

#### Ex vivo electrophysiology

To characterize intrinsic excitability, custom code in Igor Pro (Wavemetrics) was used to extract the instantaneous firing rate for each current step, rheobase, maximum firing rate, resting membrane potential, action potential threshold, spike half width, afterhyperpolatization (AHP) and voltage deflections to depolarizing and hyperpolarizing current steps. The number of action potentials for every step was manually counted. For excitability, current-response curves were compared using a two-way repeated measures ANOVA across treatment timepoints and a Geissner-Greenhouse correction, with a post-hoc Tukey test. For comparing passive and active properties across treatment timepoints, significance was determined using a Kruskal-Wallis test. A Dunn’s post hoc test was applied to test for significance between individual groups (Figure 6 and S4). For mEPSC frequency and amplitude measurements, custom code in Igor Pro (Wavemetrics) was used to extract mEPSCs events. Only cells with at least 500 detected events were included in subsequent analysis. Cumulative probability plots were generated from the first 500 mEPSC events per cell. The average amplitudes and frequencies for each cell were compared across treatment timepoints using a Kruskal-Wallis test with a Dunn’s post-hoc (Figure 5). Dopamine-evoked GIRK currents were quantified manually using Igor Pro and Matlab. A Kruskal-Wallis test with a Dunn’s post-hoc was used to compare the amplitudes and ratio of dopamine-evoked GIRK currents (Figure 7).

#### c-Fos quantification

c-Fos^+^ cells were detected from coronal striatal sections using QuPath (open-source software for image analysis, https://qupath.github.io/). Detailed methods for quantification can be found at: https://github.com/UCSF-Nelson-Lab/QuPath-scripts-for-cFos-analysis. Briefly, images were imported into QuPath. The DLS region was manually annotated for 3 sections per mouse. c-Fos^+^ cell bodies were identified and quantified using a custom script, which was adapted from the extension StarDist from QuPath. Colocalization of c-Fos^+^ cell bodies with tdTomato was performed with a custom script adapted from https://gist.github.com/Svidro/68dd668af64ad91b2f76022015dd8a45. The custom-made scripts can be found at: https://github.com/UCSF-Nelson-Lab/QuPath-scripts-for-cFos-analysis. Changes in the number of c-Fos^+^ and double c-Fos^+^/tdTomato^+^ cells across treatment timepoints were compared using a Kruskal-Wallis test with a Dunn’s post-hoc (Figure 3).

## Supplementary Material

Supplement 1 Figure S1 (related to Figure 1). Changes in mouse velocity after repetitive levodopa.
Figure S2 (related to Figure 2). More rapid LID offset relates to D2-MSN recovery kinetics.
Figure S3 (related to Figure 2). Worsening of LID onset relates to excessive increase in D1-MSN and decrease in D2-MSN activity, while faster LID resolution reflects more rapid recovery of D2-MSN activity.
Figure S4 (related to Figure 6). D2-MSN intrinsic excitability remains unchanged after repetitive levodopa.
Table S1. Experimental design and statistical analysis of all key experiments.

## Figures and Tables

**KEY RESOURCES TABLE:** 

REAGENT or RESOURCE	SOURCE	IDENTIFIER
**Antibodies**		
Rabbit anti-TH	Pel-Freez	RRID: AB_2617184; Cat# P40101-150
Alexa Fluor 488 Donkey Anti-Rabbit IgG	Jackson ImmunoResearch	RRID: AB_2340619; Cat# 711-546-152
Alexa Fluor 568 Donkey Anti-Rabbit IgG	Invitrogen	RRID: AB_2340619; Cat# 711-546-152
Alexa Fluor 647 Donkey Anti-Rabbit IgG	Jackson ImmunoResearch	RRID: AB_2534017; Cat# A10042
Rabbit anti c-Fos	Cell Signaling Technology	RRID: AB_2247211; Cat# 2250
**Bacterial and virus strains**		
AAV9-hsyn-TdTomato-T2A-mGIRK2-1-A22A-WPRE-bGH	UPenn	V4822MI-R
AAV9-hsyn-GRAB-DA2h	Yulong Li Lab	Addgene 140554; lot v188117
AAV1.Syn.Flex.GCaMP6s.WPRE.SV40	UPenn	Addgene 100845; lot CS1323
**Chemicals, peptides, and recombinant proteins**		
DiO	Invitrogen	Cat# V22889
6-Hydroxydopamine hydrobromide	Sigma-Aldrich	Cat# H116
Despiramine hydrochloride	Sigma-Aldrich	Cat# D3900
Benserazide hydrochloride	Sigma-Aldrich	Cat# B7283
3,4-Dihydroxy-L-phenylalanine	Sigma-Aldrich	Cat# D9628
Lidocaine N-ethyl chloride	Sigma-Aldrich	Cat# L1663
Cesium methanesulfonate	Sigma-Aldrich	Cat# C1426
Potassium Methanesulfonate	Sigma-Aldrich	Cat# 83000
Potassium D-Gluconate	Sigma-Aldrich	Cat# G4500
BAPTA-Na_4_	Sigma-Aldrich	Cat# 14513
Phosphocreatine disodium salt hydrate	Sigma-Aldrich	Cat# P7936
Guanosine 5’-triphosphate sodium salt	Sigma-Aldrich	Cat# G8877
Adenosine 5’-triphosphage magnesium salt	Sigma-Aldrich	Cat# A9187
Picrotoxin	Sigma-Aldrich	Cat# P1675
CNQX	Tocris	Cat# 1045
CNQX	Sigma-Aldrich	Cat# C239
DL-APV	Abcam	Cat# AB120271
Triton X-100	Sigma-Aldrich	Cat# T8787
Tetrodotoxin	Tocris	Cat# 1069
SCH23390	Tocris	Cat# 0925
Scopolamine	Tocris	Cat# 1414
CGP55485	Tocris	Cat# 1248
Dopamine	Sigma-Aldrich	Cat# H8502
**Critical commercial assays**		
VECTASHIELD Antifade Mounting Medium	Vector Laboratories	RRID: AB_2336789; Cat# H-1000
**Experimental models: Organisms/strains**		
Mouse: Wild-type C57Bl/6J	The Jackson Laboratory	RRID: IMSR_JAX:000664
Mouse: Stock Tg(Drd2-EGFP)S118Gsat/Mmnc Mus Musculus	MMRRC	RRID: MMRRC_000230-UNC
Mouse: B6.FVB(Cg)-Tg(Drd1-Cre)EY217Gsat/Mmucd	MMRRC	RRID: MMRRC_034258-UCD
Mouse: B6.FVB(Cg)-Tg(Adora2a-Cre)KG139Gsat/Mmucd	MMRRC	RRID: MMRRC_036158-UCD
Mouse: FVB.Cg-Tg(Drd1a-tdTomato)5Calak/Mmnc	MMRRC	RRID: MMRRC_030512-UNC
**Deposited data**		
Dataset and code	Zenodo	10.5281/zenodo.15446526
**Software and algorithms**		
IgorPro	Wavemetrics	RRID: SCR_00325; http://www.wavemetrics.com/products/igorpro/igorpro.htm
mafPC (software package for use with IgorPro)	Xu-Friedman Lab	https://www.xufriedman.org/mafpc
MATLAB R2015a	MathWorks	RRID: SCR_001622; https://www.mathworks.com/products/matlab/
Adobe Illustrator	Adobe	RRID: SCR_014198; http://www.adobe.com/products/illustrator
ImageJ	NIH	RRID: SCR_003070; https://imagej.nih.gov/ij/
EthovisionXT	Noldus	RRID: SCR_000441; http://www.noldus.com/animal-behavior-research/products/ethovision-xt
MAP Software (RASPUTIN 2.4)	Plexon	RRID: SCR_003170; https://plexon.com/wp-content/uploads/2017/06/RASPUTIN-Manual.pdf
Offline Sorter	Plexon	RRID: SCR_000012; http://plexon.com/products/offline-sorter
NeuroExplorer	Plexon	RRID: SCR_001818; http://www.neuroexplorer.com/
Axon MultiClamp Commander	Axon	RRID: SCR_018455; http://mdc.custhelp.com/app/answers/detail/aid/18877/~/axon%E2%84%A2-multi
NIS-Elements	Nikon	RRID: SCR_014329; https://www.microscope.healthcare.nikon.com/products/software
NeuroInfo	MBF Biosciences	RRID: SCR_017346; https://www.mbfbioscience.com/products/neuroinfo/
QuPath	QuPath	RRID: SCR_018257; https://qupath.github.io/
Synapse	TDT	N/A
Kilosort	Cortex Lab - UCL	N/A https://github.com/cortex-lab/KiloSort
Phy	Cortex Lab - UCL	N/A https://github.com/cortex-lab/phy
IC Capture	The Imaging Source	N/A
Boris	Friard and Gamba, 2016^85^	N/A boris.unito.it
**Other**		
32 Channel Fixed Optrode Array	Innovative Neurophysiology	Custom; http://www.inphysiology.com/optogenetic-applications/
64 Channel Silicon probe	Cambridge Neurotech	Cat# H6
400 μm optic fibers	Doric	MFC_400/430-0.66_4mm_MF1.25_FLT
10-Channel Slip Ring Electrical Commutator	Dragonfly	Model SL-10-C; https://campdeninstruments.com/products/10-ch-slip-ring-commuta
Master-8	AMPI	http://www.ampi.co.il/master8cp.html
M-Series (M60) Amplifier System for Multiplexing	Triangle BioSystems	http://www.trianglebiosystems.com/m-series-systems.html
Single Channel Temperature Controller	Warner Instruments	Cat#TC-324C
MINIPULS 3 Peristaltic Pumps	Gilson	Cat#F155008
X-Cite 120LED Boost	Excelitas	Cat#010-00326R
ITC-18 16-bit Multi-Channel Data Acquisition Interface	Heka	RRID: SCR_023164
Multiclamp 700B Microelectrode Amplifier	Molecular Devices	RRID: SCR_018455
RHD USB Interface Board	Intan Technologies	Cat# C3100
Assisted Electrical Rotary Joint	Doric	AERJ_24_PZN12
Video camera	The Imaging Source	DMK 33UX273
Dataset	Zenodo	10.5281/zenodo.15446526

## References

[1] Goetz C.G. (2011). The history of Parkinson’s disease: early clinical descriptions and neurological therapies. Cold Spring Harb Perspect Med 1, a008862. 10.1101/cshperspect.a008862. 22229124 PMC3234454

[2] Kalia L. V, and Lang A.E. (2015). Parkinson’s disease. Lancet 386, 896–912. 10.1016/S0140-6736(14)61393–3. 25904081

[3] Carta M., and Bezard E. (2011). Contribution of pre-synaptic mechanisms to L-DOPA-induced dyskinesia. Neuroscience 198, 245–251. 10.1016/j.neuroscience.2011.07.070. 21840375

[4] Connolly B.S., and Lang A.E. (2014). Pharmacological treatment of Parkinson disease: a review. JAMA 311, 1670–1683. 10.1001/jama.2014.3654. 24756517

[5] Cenci M.A. (2014). Presynaptic Mechanisms of l-DOPA-Induced Dyskinesia: The Findings, the Debate, and the Therapeutic Implications. Front Neurol 5, 242. 10.3389/fneur.2014.00242. 25566170 PMC4266027

[6] Jankovic J. (2005). Motor fluctuations and dyskinesias in Parkinson’s disease: clinical manifestations. Mov Disord 20 Suppl 1, S11–6. 10.1002/mds.20458. 15822109

[7] Cenci M.A., Riggare S., Pahwa R., Eidelberg D., and Hauser R.A. (2020). Dyskinesia matters. Mov Disord 35, 392–396. 10.1002/mds.27959. 31872501

[8] Espay A.J., Morgante F., Merola A., Fasano A., Marsili L., Fox S.H., Bezard E., Picconi B., Calabresi P., and Lang A.E. (2018). Levodopa-induced dyskinesia in Parkinson disease: Current and evolving concepts. Ann Neurol 84, 797–811. 10.1002/ana.25364. 30357892

[9] Manson A., Stirpe P., and Schrag A. (2012). Levodopa-Induced-Dyskinesias Clinical Features, Incidence, Risk Factors, Management and Impact on Quality of Life. J Parkinsons Dis 2, 189–198. 10.3233/JPD-2012-120103. 23938226

[10] Ryan M.B., Bair-Marshall C., and Nelson A.B. (2018). Aberrant Striatal Activity in Parkinsonism and Levodopa-Induced Dyskinesia. Cell Rep 23, 3438–3446.e5. 10.1016/j.celrep.2018.05.059. 29924988 PMC6407866

[11] Twedell E.L., Bair-Marshall C.J., Girasole A.E., Scaria L.K., Sridhar S., and Nelson A.B. (2024). Striatal lateral inhibition regulates action selection in a mouse model of levodopa-induced dyskinesia. Preprint, 10.1101/2024.10.11.617939 https://doi.org/10.1101/2024.10.11.617939.

[12] Girasole A.E., Lum M.Y., Nathaniel D., Bair-Marshall C.J., Guenthner C.J., Luo L., Kreitzer A.C., and Nelson A.B. (2018). A Subpopulation of Striatal Neurons Mediates Levodopa-Induced Dyskinesia. Neuron 97, 787–795.e6. 10.1016/j.neuron.2018.01.017. 29398356 PMC6233726

[13] Ryan M.B., Girasole A.E., Flores A.J., Twedell E.L., McGregor M.M., Brakaj R., Paletzki R.F., Hnasko T.S., Gerfen C.R., and Nelson A.B. (2024). Excessive firing of dyskinesia-associated striatal direct pathway neurons is gated by dopamine and excitatory synaptic input. Cell Rep 43, 114483. 10.1016/j.celrep.2024.114483. 39024096 PMC12080562

[14] Fieblinger T., Graves S.M., Sebel L.E., Alcacer C., Plotkin J.L., Gertler T.S., Chan C.S., Heiman M., Greengard P., Cenci M.A., (2014). Cell type-specific plasticity of striatal projection neurons in parkinsonism and L-DOPA-induced dyskinesia. Nat Commun 5, 5316. 10.1038/ncomms6316. 25360704 PMC4431763

[15] Shen W., Plotkin J.L., Francardo V., Ko W.K.D., Xie Z., Li Q., Fieblinger T., Wess J., Neubig R.R., Lindsley C.W., (2015). M4 Muscarinic Receptor Signaling Ameliorates Striatal Plasticity Deficits in Models of L-DOPA-Induced Dyskinesia. Neuron 88, 762–773. 10.1016/j.neuron.2015.10.039. 26590347 PMC4864040

[16] Zhai S., Cui Q., Wokosin D., Sun L., Tkatch T., Crittenden J.R., Graybiel A.M., and Surmeier D.J. (2025). State-dependent modulation of spiny projection neurons controls levodopa-induced dyskinesia in a mouse model of Parkinson’s disease. Preprint, 10.1101/2025.01.02.631090 https://doi.org/10.1101/2025.01.02.631090. PMC12674127 41337591

[17] Shen W., Zhai S., Francardo V., Cui Q., Xie Z., Tkatch T., Cenci M.A., and Surmeier D.J. (2024). Cell- and state-specific plasticity of striatal glutamatergic synapses is critical to the expression of levodopa-induced dyskinesia. Preprint, 10.1101/2024.06.14.599055 https://doi.org/10.1101/2024.06.14.599055. PMC13252713 42086052

[18] Murer M.G., and Moratalla R. (2011). Striatal Signaling in L-DOPA-Induced Dyskinesia: Common Mechanisms with Drug Abuse and Long Term Memory Involving D1 Dopamine Receptor Stimulation. Front Neuroanat 5, 51. 10.3389/fnana.2011.00051. 21886608 PMC3154293

[19] Paz R.M., Tubert C., Stahl A.M., Amarillo Y., Rela L., and Murer M.G. (2021). Levodopa Causes Striatal Cholinergic Interneuron Burst-Pause Activity in Parkinsonian Mice. Mov Disord. 10.1002/mds.28516. 33547844

[20] Sáez M., Keifman E., Alberquilla S., Coll C., Reig R., Murer M.G., and Moratalla R. (2023). D2 dopamine receptors and the striatopallidal pathway modulate L-DOPA-induced dyskinesia in the mouse. Neurobiol Dis 186, 106278. 10.1016/j.nbd.2023.106278. 37683958

[21] Keifman E., Ruiz-DeDiego I., Pafundo D.E., Paz R.M., Solís O., Murer M.G., and Moratalla R. (2019). Optostimulation of striatonigral terminals in substantia nigra induces dyskinesia that increases after L-DOPA in a mouse model of Parkinson’s disease. Br J Pharmacol 176, 2146–2161. 10.1111/bph.14663. 30895594 PMC6555865

[22] Singh A., and Papa S.M. (2020). Striatal Oscillations in Parkinsonian Non-Human Primates. Neuroscience 449, 116–122. 10.1016/j.neuroscience.2020.09.004. 32905842 PMC7642120

[23] Pagano G., Yousaf T., and Politis M. (2017). PET Molecular Imaging Research of Levodopa-Induced Dyskinesias in Parkinson’s Disease. Curr Neurol Neurosci Rep 17, 90. 10.1007/s11910-017-0794-2. PMC5626800 28975571

[24] Gerfen C.R., Engber T.M., Mahan L.C., Susel Z., Chase T.N., Monsma F.J.J., and Sibley D.R. (1990). D1 and D2 dopamine receptor-regulated gene expression of striatonigral and striatopallidal neurons. Science 250, 1429–1432. 10.1126/science.2147780. 2147780

[25] Cui G., Jun S.B., Jin X., Pham M.D., Vogel S.S., Lovinger D.M., and Costa R.M. (2013). Concurrent activation of striatal direct and indirect pathways during action initiation. Nature 494, 238–242. 10.1038/nature11846. 23354054 PMC4039389

[26] Jin X., and Costa R.M. (2010). Start/stop signals emerge in nigrostriatal circuits during sequence learning. Nature 466, 457–462. 10.1038/nature09263. 20651684 PMC3477867

[27] Tecuapetla F., Jin X., Lima S.Q., and Costa R.M. (2016). Complementary Contributions of Striatal Projection Pathways to Action Initiation and Execution. Cell 166, 703–715. 10.1016/j.cell.2016.06.032. 27453468

[28] Parker J.G., Marshall J.D., Ahanonu B., Wu Y.-W., Kim T.H., Grewe B.F., Zhang Y., Li J.Z., Ding J.B., Ehlers M.D., (2018). Diametric neural ensemble dynamics in parkinsonian and dyskinetic states. Nature 557, 177–182. 10.1038/s41586-018-0090-6. 29720658 PMC6526726

[29] Alcacer C., Klaus A., Mendonça M., Abalde S.F., Cenci M.A., and Costa R.M. (2024). Abnormal hyperactivity of specific striatal ensembles encodes distinct dyskinetic behaviors revealed by high-resolution clustering. Preprint, 10.1101/2024.09.06.611664 https://doi.org/10.1101/2024.09.06.611664. 40638389

[30] Alcacer C., Andreoli L., Sebastianutto I., Jakobsson J., Fieblinger T., and Cenci M.A. (2017). Chemogenetic stimulation of striatal projection neurons modulates responses to Parkinson’s disease therapy. J Clin Invest 127, 720–734. 10.1172/JCI90132. 28112685 PMC5272195

[31] Castela I., Casado-Polanco R., Rubio Y.V.-W., da Silva J.A., Marquez R., Pro B., Moratalla R., Redgrave P., Costa R.M., Obeso J., (2023). Selective activation of striatal indirect pathway suppresses levodopa induced-dyskinesias. Neurobiol Dis 176, 105930. 10.1016/j.nbd.2022.105930. 36414182

[32] Perez X.A., Zhang D., Bordia T., and Quik M. (2017). Striatal D1 medium spiny neuron activation induces dyskinesias in parkinsonian mice. Movement Disorders 32, 538–548. 10.1002/mds.26955. 28256010 PMC5398928

[33] Hernández F., L., Castela, I., Ruiz-DeDiego, I., Obeso, J.A., and Moratalla, R. (2017). Striatal activation by optogenetics induces dyskinesias in the 6-hydroxydopamine rat model of Parkinson disease. Movement Disorders 32, 530–537. 10.1002/mds.26947. 28256089

[34] Lahiri A.K., and Bevan M.D. (2020). Dopaminergic Transmission Rapidly and Persistently Enhances Excitability of D1 Receptor-Expressing Striatal Projection Neurons. Neuron 106, 277–290.e6. 10.1016/j.neuron.2020.01.028. 32075716 PMC7182485

[35] Tsuboi D., Otsuka T., Shimomura T., Faruk M.O., Yamahashi Y., Amano M., Funahashi Y., Kuroda K., Nishioka T., Kobayashi K., (2022). Dopamine drives neuronal excitability via KCNQ channel phosphorylation for reward behavior. Cell Rep 40, 111309. 10.1016/j.celrep.2022.111309. 36070693

[36] Shen W., Flajolet M., Greengard P., and Surmeier D.J. (2008). Dichotomous Dopaminergic Control of Striatal Synaptic Plasticity. Science (1979) 321, 848–851. 10.1126/science.1160575. 18687967 PMC2833421

[37] COSTA R.M. (2007). Plastic Corticostriatal Circuits for Action Learning. Ann N Y Acad Sci 1104, 172–191. 10.1196/annals.1390.015. 17435119

[38] Calabresi P., Picconi B., Tozzi A., and Di Filippo M. (2007). Dopamine-mediated regulation of corticostriatal synaptic plasticity. Trends Neurosci 30, 211–219. 10.1016/j.tins.2007.03.001. 17367873

[39] Wang Z., Kai L., Day M., Ronesi J., Yin H.H., Ding J., Tkatch T., Lovinger D.M., and Surmeier D.J. (2006). Dopaminergic control of corticostriatal long-term synaptic depression in medium spiny neurons is mediated by cholinergic interneurons. Neuron 50, 443–452. 10.1016/j.neuron.2006.04.010. 16675398

[40] Jin X., and Costa R.M. (2015). Shaping action sequences in basal ganglia circuits. Curr Opin Neurobiol 33, 188–196. 10.1016/j.conb.2015.06.011. 26189204 PMC4523429

[41] Escande M. V, Taravini I.R.E., Zold C.L., Belforte J.E., and Murer M.G. (2016). Loss of Homeostasis in the Direct Pathway in a Mouse Model of Asymptomatic Parkinson’s Disease. J Neurosci 36, 5686–5698. 10.1523/JNEUROSCI.0492-15.2016. 27225760 PMC6601837

[42] Fieblinger T., Zanetti L., Sebastianutto I., Breger L.S., Quintino L., Lockowandt M., Lundberg C., and Cenci M.A. (2018). Striatonigral neurons divide into two distinct morphological-physiological phenotypes after chronic L-DOPA treatment in parkinsonian rats. Sci Rep 8, 10068. 10.1038/s41598-018-28273-5. 29968767 PMC6030109

[43] Gomez G., Escande M. V., Suarez L.M., Rela L., Belforte J.E., Moratalla R., Murer M.G., Gershanik O.S., and Taravini I.R.E. (2019). Changes in Dendritic Spine Density and Inhibitory Perisomatic Connectivity onto Medium Spiny Neurons in l-Dopa-Induced Dyskinesia. Mol Neurobiol 56, 6261–6275. 10.1007/s12035-019-1515-4. 30746639

[44] Suárez L.M., Solís O., Caramés J.M., Taravini I.R., Solís J.M., Murer M.G., and Moratalla R. (2014). L-DOPA treatment selectively restores spine density in dopamine receptor D2-expressing projection neurons in dyskinetic mice. Biol Psychiatry 75, 711–722. 10.1016/j.biopsych.2013.05.006. 23769604

[45] Picconi B., Centonze D., Håkansson K., Bernardi G., Greengard P., Fisone G., Cenci M.A., and Calabresi P. (2003). Loss of bidirectional striatal synaptic plasticity in L-DOPA-induced dyskinesia. Nat Neurosci 6, 501–506. 10.1038/nn1040. 12665799

[46] Cenci M.A., and Lundblad M. (2007). Ratings of L-DOPA-Induced Dyskinesia in the Unilateral 6-OHDA Lesion Model of Parkinson’s Disease in Rats and Mice. Curr Protoc Neurosci 41. 10.1002/0471142301.ns0925s41. 18428668

[47] Santini E., Alcacer C., Cacciatore S., Heiman M., Hervé D., Greengard P., Girault J.-A., Valjent E., and Fisone G. (2009). L-DOPA activates ERK signaling and phosphorylates histone H3 in the striatonigral medium spiny neurons of hemiparkinsonian mice. J Neurochem 108, 621–633. 10.1111/j.1471-4159.2008.05831.x. 19187092

[48] Cenci M.A., Jörntell H., and Petersson P. (2018). On the neuronal circuitry mediating L-DOPA-induced dyskinesia. J Neural Transm (Vienna) 125, 1157–1169. 10.1007/S00702-018-1886-0. 29704061 PMC6060876

[49] Berton O., Guigoni C., Li Q., Bioulac B.H., Aubert I., Gross C.E., DiLeone R.J., Nestler E.J., and Bezard E. (2009). Striatal Overexpression of ΔJunD Resets L-DOPA-Induced Dyskinesia in a Primate Model of Parkinson Disease. Biol Psychiatry 66, 554–561. 10.1016/j.biopsych.2009.04.005. 19481198 PMC2825375

[50] Pavón N., Martín A.B., Mendialdua A., and Moratalla R. (2006). ERK phosphorylation and FosB expression are associated with L-DOPA-induced dyskinesia in hemiparkinsonian mice. Biol Psychiatry 59, 64–74. 10.1016/j.biopsych.2005.05.044. 16139809

[51] Santini E., Valjent E., Usiello A., Carta M., Borgkvist A., Girault J.-A., Hervé D., Greengard P., and Fisone G. (2007). Critical involvement of cAMP/DARPP-32 and extracellular signal-regulated protein kinase signaling in L-DOPA-induced dyskinesia. J Neurosci 27, 6995–7005. 10.1523/JNEUROSCI.0852-07.2007. 17596448 PMC6672217

[52] Gage G.J., Stoetzner C.R., Wiltschko A.B., and Berke J.D. (2010). Selective Activation of Striatal Fast-Spiking Interneurons during Choice Execution. Neuron 67, 466–479. 10.1016/j.neuron.2010.06.034. 20696383 PMC2920892

[53] Harris K.D., Henze D.A., Csicsvari J., Hirase H., and Buzsáki G. (2000). Accuracy of Tetrode Spike Separation as Determined by Simultaneous Intracellular and Extracellular Measurements. J Neurophysiol 84, 401–414. 10.1152/jn.2000.84.1.401. 10899214

[54] Berke J.D., Okatan M., Skurski J., and Eichenbaum H.B. (2004). Oscillatory Entrainment of Striatal Neurons in Freely Moving Rats. Neuron 43, 883–896. 10.1016/j.neuron.2004.08.035. 15363398

[55] Sun F., Zhou J., Dai B., Qian T., Zeng J., Li X., Zhuo Y., Zhang Y., Wang Y., Qian C., (2020). Next-generation GRAB sensors for monitoring dopaminergic activity in vivo. Nat Methods 17, 1156–1166. 10.1038/s41592-020-00981-9. 33087905 PMC7648260

[56] Wickens J.R., Reynolds J.N., and Hyland B.I. (2003). Neural mechanisms of reward-related motor learning. Curr Opin Neurobiol 13, 685–690. 10.1016/j.conb.2003.10.013. 14662369

[57] Surmeier D.J., Bargas J., Hemmings H.C., Nairn A.C., and Greengard P. (1995). Modulation of calcium currents by a D1 dopaminergic protein kinase/phosphatase cascade in rat neostriatal neurons. Neuron 14, 385–397. 10.1016/0896-6273(95)90294–5. 7531987

[58] Shen W., Hernandez-Lopez S., Tkatch T., Held J.E., and Surmeier D.J. (2004). Kv1.2-containing K+ channels regulate subthreshold excitability of striatal medium spiny neurons. J Neurophysiol 91, 1337–1349. 10.1152/jn.00414.2003. 13679409

[59] Pineda J.C., Galarraga E., Bargas J., Cristancho M., and Aceves J. (1992). Charybdotoxin and apamin sensitivity of the calcium-dependent repolarization and the afterhyperpolarization in neostriatal neurons. J Neurophysiol 68, 287–294. 10.1152/jn.1992.68.1.287. 1381420

[60] Pérez-Garci E., Bargas J., and Galarraga E. (2003). The role of Ca2+ channels in the repetitive firing of striatal projection neurons. Neuroreport 14, 1253–1256. 10.1097/00001756-200307010-00013. 12824770

[61] Vilchis C., Bargas J., Ayala G.X., Galván E., and Galarraga E. (1999). Ca2+ channels that activate Ca2+-dependent K+ currents in neostriatal neurons. Neuroscience 95, 745–752. 10.1016/S0306-4522(99)00493–5. 10670441

[62] Shen W., Tian X., Day M., Ulrich S., Tkatch T., Nathanson N.M., and Surmeier D.J. (2007). Cholinergic modulation of Kir2 channels selectively elevates dendritic excitability in striatopallidal neurons. Nat Neurosci 10, 1458–1466. 10.1038/nn1972. 17906621

[63] Pacheco-Cano M.T., Bargas J., Hernández-López S., Tapia D., and Galarraga E. (1996). Inhibitory action of dopamine involves a subthreshold Cs(+)-sensitive conductance in neostriatal neurons. Exp Brain Res 110, 205–211. 10.1007/BF00228552. 8836685

[64] Zhao B., Zhu J., Dai D., Xing J., He J., Fu Z., Zhang L., Li Z., and Wang W. (2016). Differential dopaminergic regulation of inwardly rectifying potassium channel mediated subthreshold dynamics in striatal medium spiny neurons. Neuropharmacology 107, 396–410. 10.1016/j.neuropharm.2016.03.037. 27018450

[65] Nisenbaum E.S., Xu Z.C., and Wilson C.J. (1994). Contribution of a slowly inactivating potassium current to the transition to firing of neostriatal spiny projection neurons. J Neurophysiol 71, 1174–1189. 10.1152/jn.1994.71.3.1174. 8201411

[66] Marcott P.F., Mamaligas A.A., and Ford C.P. (2014). Phasic Dopamine Release Drives Rapid Activation of Striatal D2-Receptors. Neuron 84, 164–176. 10.1016/j.neuron.2014.08.058. PMC4325987 25242218

[67] Gong S., Fayette N., Heinsbroek J.A., and Ford C.P. (2021). Cocaine shifts dopamine D2 receptor sensitivity to gate conditioned behaviors. Neuron 109, 3421–3435.e5. 10.1016/j.neuron.2021.08.012. 34506723 PMC8571051

[68] Darmopil S., Martín A.B., De Diego I.R., Ares S., and Moratalla R. (2009). Genetic inactivation of dopamine D1 but not D2 receptors inhibits L-DOPA-induced dyskinesia and histone activation. Biol Psychiatry 66, 603–613. 10.1016/j.biopsych.2009.04.025. 19520364

[69] Lindgren H.S., Andersson D.R., Lagerkvist S., Nissbrandt H., and Cenci M.A. (2010). l-DOPA-induced dopamine efflux in the striatum and the substantia nigra in a rat model of Parkinson’s disease: temporal and quantitative relationship to the expression of dyskinesia. J Neurochem 112, 1465–1476. 10.1111/j.1471-4159.2009.06556.x. 20050978

[70] Park J., Kang S., Lee Y., Choi J.-W., and Oh Y.-S. (2024). Continuous long-range measurement of tonic dopamine with advanced FSCV for pharmacodynamic analysis of levodopa-induced dyskinesia in Parkinson’s disease. Front Bioeng Biotechnol 12. 10.3389/fbioe.2024.1335474. PMC10847580 38328444

[71] Vu M.-A.T., Brown E.H., Wen M.J., Noggle C.A., Zhang Z., Monk K.J., Bouabid S., Mroz L., Graham B.M., Zhuo Y., (2024). Targeted micro-fiber arrays for measuring and manipulating localized multi-scale neural dynamics over large, deep brain volumes during behavior. Neuron 112, 909–923.e9. 10.1016/j.neuron.2023.12.011. 38242115 PMC10957316

[72] Patriarchi T., Cho J.R., Merten K., Howe M.W., Marley A., Xiong W.-H., Folk R.W., Broussard G.J., Liang R., Jang M.J., (2018). Ultrafast neuronal imaging of dopamine dynamics with designed genetically encoded sensors. Science (1979) 360. 10.1126/science.aat4422. PMC6287765 29853555

[73] Planert H., Berger T.K., and Silberberg G. (2013). Membrane Properties of Striatal Direct and Indirect Pathway Neurons in Mouse and Rat Slices and Their Modulation by Dopamine. PLoS One 8, e57054. 10.1371/journal.pone.0057054. 23469183 PMC3585935

[74] Legaria A.A., Matikainen-Ankney B.A., Yang B., Ahanonu B., Licholai J.A., Parker J.G., and Kravitz A. V. (2022). Fiber photometry in striatum reflects primarily nonsomatic changes in calcium. Nat Neurosci 25, 1124–1128. 10.1038/s41593-022-01152-z. 36042311 PMC10152879

[75] Tepper J.M., Koós T., and Wilson C.J. (2004). GABAergic microcircuits in the neostriatum. Trends Neurosci 27, 662–669. 10.1016/j.tins.2004.08.007. 15474166

[76] Tepper J.M., Koós T., Ibanez-Sandoval O., Tecuapetla F., Faust T.W., and Assous M. (2018). Heterogeneity and Diversity of Striatal GABAergic Interneurons: Update 2018. Front Neuroanat 12, 91. 10.3389/fnana.2018.00091. 30467465 PMC6235948

[77] Taverna S., Ilijic E., and Surmeier D.J. (2008). Recurrent Collateral Connections of Striatal Medium Spiny Neurons Are Disrupted in Models of Parkinson’s Disease. The Journal of Neuroscience 28, 5504–5512. 10.1523/JNEUROSCI.5493-07.2008. 18495884 PMC3235738

[78] Gittis A.H., Hang G.B., LaDow E.S., Shoenfeld L.R., Atallah B.V., Finkbeiner S., and Kreitzer A.C. (2011). Rapid Target-Specific Remodeling of Fast-Spiking Inhibitory Circuits after Loss of Dopamine. Neuron 71, 858–868. 10.1016/j.neuron.2011.06.035. 21903079 PMC3170520

[79] Paz R.M., and Murer M.G. (2021). Mechanisms of Antiparkinsonian Anticholinergic Therapy Revisited. Neuroscience 467, 201–217. 10.1016/j.neuroscience.2021.05.026. 34048797

[80] Shen W., Hamilton S.E., Nathanson N.M., and Surmeier D.J. (2005). Cholinergic Suppression of KCNQ Channel Currents Enhances Excitability of Striatal Medium Spiny Neurons. Journal of Neuroscience 25, 7449–7458. 10.1523/JNEUROSCI.1381-05.2005. 16093396 PMC6725301

[81] Nielsen B.E., and Ford C.P. (2024). Reduced striatal M4-cholinergic signaling following dopamine loss contributes to parkinsonian and <scp>l</scp> -DOPA–induced dyskinetic behaviors. Sci Adv 10. 10.1126/sciadv.adp6301. PMC11578179 39565858

[82] Abudukeyoumu N., Hernandez-Flores T., Garcia-Munoz M., and Arbuthnott G.W. (2019). Cholinergic modulation of striatal microcircuits. Eur J Neurosci 49, 604–622. 10.1111/ejn.13949. 29797362 PMC6587740

[83] A. Cenci M., E. Ohlin K., and Odin P. (2011). Current Options and Future Possibilities for the Treatment of Dyskinesia and Motor Fluctuations in Parkinson’s Disease. CNS Neurol Disord Drug Targets 10, 670–684. 10.2174/187152711797247885. 21838677

[84] Goggi J.L., Sardini A., Egerton A., Strange P.G., and Grasby P.M. (2007). Agonist-dependent internalization of D2 receptors: Imaging quantification by confocal microscopy. Synapse 61, 231–241. 10.1002/syn.20360. 17230553

[85] Friard O., and Gamba M. (2016). BORIS: a free, versatile open-source event-logging software for video/audio coding and live observations. Methods Ecol Evol 7, 1325–1330. 10.1111/2041-210X.12584.

