# Supplementary material for: Repetitive Levodopa Treatment Drives Cell Type-Specific Striatal Adaptations Associated With Progressive Dyskinesia in Parkinsonian Mice
Source: bioRxiv. 2025 May 21:2025.05.16.654598. Preprint. [Version 1] doi: 10.1101/2025.05.16.654598 (PMC12139814; doi:10.1101/2025.05.16.654598)
Supplement: Supplement 1 — Figure S1 (related to Figure 1). Changes in mouse velocity after repetitive levodopa. Figure S2 (related to Figure 2). More rapid LID offset relates to D2-MSN recovery kinetics. Figure S3 (related to Figure 2). Worsening of LID onset relates to excessive increase in D1-MSN and decrease in D2-MSN activity, while faster LID resolution reflects more rapid recovery of D2-MSN activity. Figure S4 (related to Figure 6). D2-MSN intrinsic excitability remains unchanged after repetitive levodopa. Table S1. Experimental design and statistical analysis of all key experiments. [file media-1.pdf]

SUPPLEMENTAL INFORMATION

Figure S1

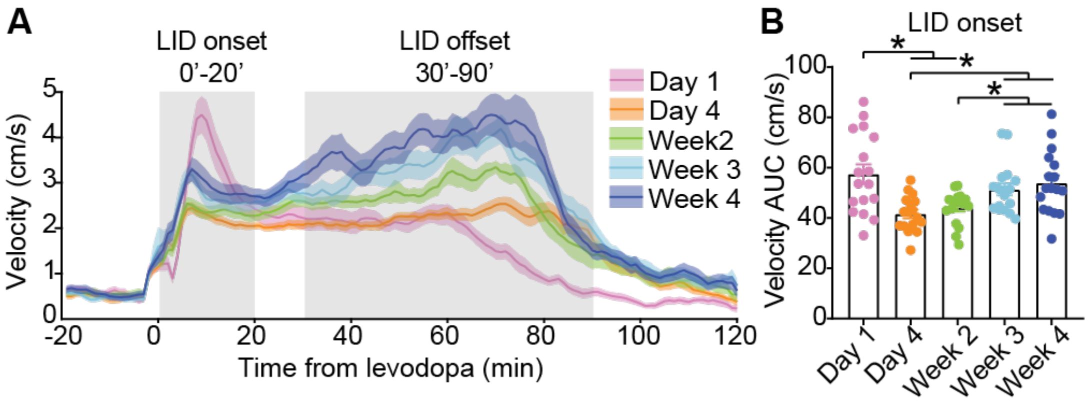

Figure S1 (related to Figure 1). Changes in locomotor velocity after repetitive levodopa. (A) Average locomotor velocity in response to IP injection of 5mg/kg levodopa for different treatment timepoints. **(B-C)** Area under the curve (AUC) of velocity traces during LID onset (B), and offset (C) phases. [RM one-way ANOVA, B: p=0.0004, post-hoc *p<0.05; C: p<0.0001, post-hoc *p<0.05]. Data shown as mean±SEM. N=17.

Figure S2

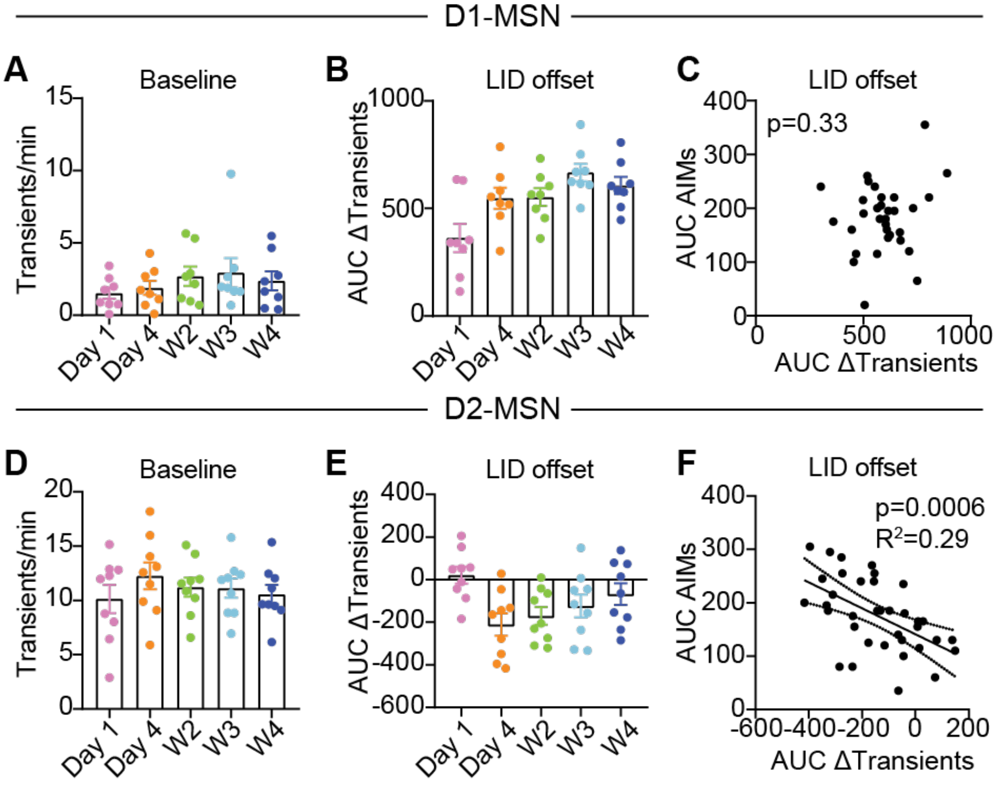

Figure S2 (related to Figure 2). More rapid LID offset relates to the recovery of D2-MSN activity. (A) Average baseline rates of D1-MSN GCaMP transients 30 min before levodopa injection. [RM one-way ANOVA, p=0.2270]. **(B)** Area under the curve of D1-MSN ∆Transients during LID offset. [RM one-way ANOVA, ns]. **(C)** Correlation of D1-MSN ∆Transients with AIMs during LID offset. **(D)** Average baseline rates of D2-MSN GCaMP transients 30 min before levodopa injection. [RM one-way ANOVA, p=0.1573]. **(E)** Area under the curve of D2-MSN ∆Transients during LID offset. [RM one-way ANOVA, p=0.0436]. **(F)** Correlation of D2-MSN ∆Transients with AIMs during LID offset. N=8 D1-Cre and N=9 A2a-Cre. Each dot is one mouse. Data shown as mean±SEM (A, C).

Figure S3

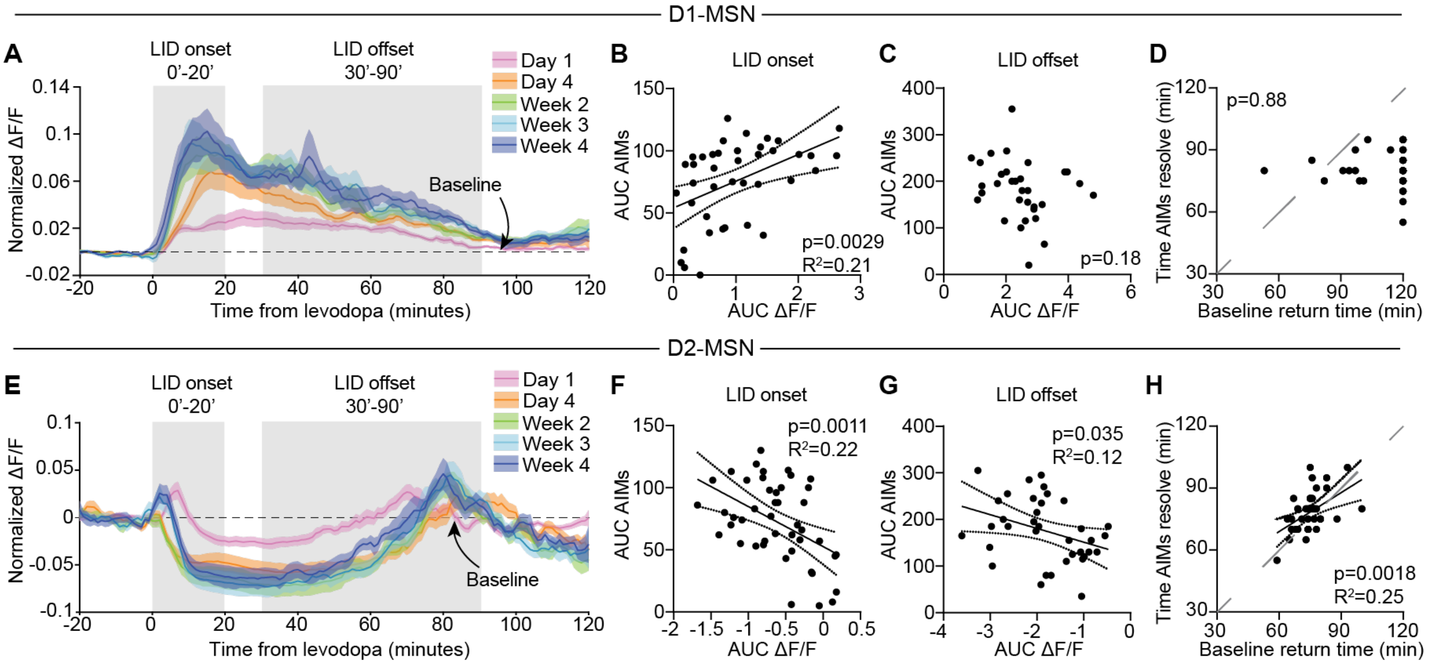

Figure S3 (related to Figure 2). Changes in striatal activity parallel alterations in dyskinesia during repetitive levodopa treatment. GCaMP fiber photometry in DLS D1-MSNs (A-D) or D2-MSNs (E-H) of 6-OHDA mice treated with levodopa. **(A)** D1-MSN ΔF/F response to levodopa with baseline subtraction across treatment timepoints. LID onset and offset phases are shown in grey. **(B-C)** Correlation between AIMs and D1-MSN ΔF/F during LID onset (B) and offset (C) phases. **(D)** Correlation between AIMs resolution time and D1-MSN ΔF/F return to baseline. **(E)** D2-MSN ΔF/F response to levodopa with baseline subtraction across treatment timepoints. **(F-G)** Correlation between AIMs and D2-MSN ΔF/F during LID onset (F) and offset (G) phases. **(H)** Correlation between AIMs resolution time and D2-MSN ΔF/F return to baseline. N=9 A2a-Cre and N=8 D1-Cre mice. Data shown as mean±SEM (A, E).

Figure S4

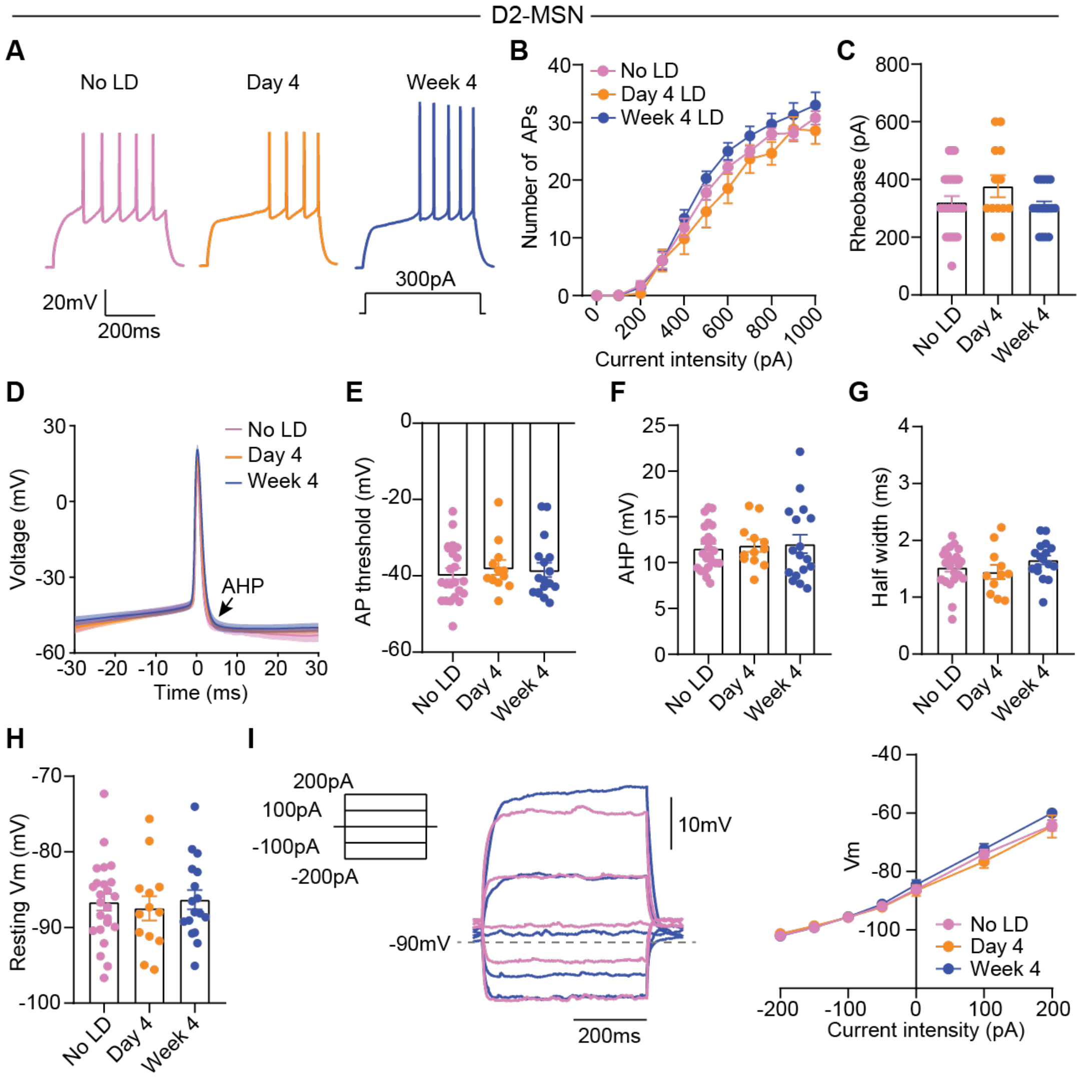

Figure S4 (related to Figure 6). D2-MSN intrinsic excitability remains unchanged after repetitive levodopa. (A) Voltage responses to current injection in D2-MSN after different levodopa treatment durations. **(B)** Number of action potentials (APs) in response to depolarizing current steps. [Two-way ANOVA, ns]. **(C)** D2-MSN rheobase across treatment duration. [Kruskal-Wallis, ns]. **(D)** Average action potential shape. **(E-H)** Action potential threshold (E), afterhyperpolarization (AHP, F), half width (G) and resting membrane potential (H). [Kruskal-Wallis, ns]. **(I)** Voltage deflection to current injections. [Two-way ANOVA, ns]. No LD: n=23-24, N=11; Day 4: n=12-13, N=6; Week 4: n=17, N=5. Data shown as mean±SEM. n=cells; N=mice. Each dot represents one cell.

Table S1. Experimental design and statistical analysis of all key experiments

Experiment	Fig	Test	N (mice)	n (cells)	p-value	Significant post-hoc	p-value	Planned sample size
AIM AUC 0-20 min	1c	RM one-way ANOVA	17	-	<0.0001	Day 1 vs rest	<0.0001	N = 10 mice
AIM AUC 30-90 min	1d	RM one-way ANOVA	17	-	<0.0001	Day 4 vs week 2<br>Day 4 vs week 3<br>Day 4 vs week 4<br>Week 2 vs week 3<br>Week 2 vs week 4	0.0486<br><0.0001<br><0.0001<br>0.0285<br>0.0205	N = 10 mice
Time AIMs resolve	1e	RM one-way ANOVA	17	-	<0.0001	Day 4 vs week 2<br>Day 4 vs week 3<br>Day 4 vs week 4	0.0043<br>0.0001<br>0.0002	N = 10 mice
Velocity AUC 1-20 min	S1b	RM one-way ANOVA	17	-	0.0004	Day 1 vs Day 4<br>Day 1 vs week 2<br>Day 4 vs week 3<br>Day 4 vs week 4<br>Week 2 vs week 3<br>Week 2 vs week 4	0.0095<br>0.0179<br>0.0088<br>0.0010<br>0.0243<br>0.0111	N = 10 mice
Velocity AUC 30-90 min	S1c	RM one-way ANOVA	17	-	<0.0001	Day 4 vs week 2<br>Day 4 vs week 3<br>Day 4 vs week 4<br>Week 2 vs week 3<br>Week 2 vs week 4	0.0020<br>0.0008<br><0.0001<br>0.0150<br>0.0021	N = 10 mice
D1 AUC ∆Transients for LID onset	2f	RM one-way ANOVA	8	-	0.0003	Day 1 vs day 4<br>Day 1 vs week 2<br>Day 1 vs week 3<br>Day 1 vs week 4	0.0466<br>0.0031<br>0.0010<br>0.0004	N = 10 mice
Time D1 ∆Transients return to baseline	2g	RM one-way ANOVA	8	-	0.5063	-	-	N = 10 mice
D1 Correlation AIMs vs ∆Transients for LID onset	2h	Pearson	8 mice x 5 timepoints	-	0.0008<br>R2=0.26	-	-	N = 10 mice
D1 correlation Time AIMs resolve vs ∆Transients return to baseline	2i	Pearson	8 mice x 4 timepoints	-	0.4819	-	-	N = 10 mice
D2 AUC ∆Transients for LID onset	2l	RM one-way ANOVA	9	-	0.0001	Day 1 vs day 4<br>Day 1 vs week 2<br>Day 1 vs week 3	0.0026<br>0.0040<br>0.0342	N = 10 mice
Time D2 ∆Transients return to baseline	2m	RM one-way ANOVA	9	-	0.0047	Day 4 vs week 4	0.0344	N = 10 mice
D2 Correlation AIMs vs ∆Transients for LID onset	2n	Pearson	9 mice x 5 timepoints	-	<0.0001<br>R2=0.39	-	-	N = 10 mice
D2 correlation Time AIMs resolve vs ∆Transients return to baseline	2o	Pearson	9 mice x 4 timepoints	-	<0.0001<br>R2=0.43	-	-	N = 10 mice
D1 baseline transient frequency	S2a	RM one-way ANOVA	8	-	0.2270	-	-	N = 10 mice
D1 AUC ∆Transients for	S2b	RM one-way	8	-	0.0471	-	-	N = 10 mice

LID offset		ANOVA						
D1 Correlation AIMs vs ΔTransients for LID offset	S2c	Pearson	8 mice x 4 timepoints	-	0.3314	-	-	N = 10 mice
D2 baseline transient frequency	S2d	RM one-way ANOVA	9	-	0.1573	-	-	N = 10 mice
D2 AUC ΔTransients for LID offset	S2e	RM one-way ANOVA	9	-	0.0436	-	-	N = 10 mice
D2 Correlation AIMs vs ΔTransients for LID offset	S2f	Pearson	9 mice x 4 timepoints	-	0.0006 $R2=0.29$	-	-	N = 10 mice
D1 Correlation AIMs vs ΔF/F for LID onset	S3b	Pearson	8 mice x 5 timepoints	-	0.0029 $R2=0.21$	-	-	N = 10 mice
D1 Correlation AIMs vs ΔF/F for LID offset	S3c	Pearson	8 mice x 4 timepoints	-	0.1833	-	-	N = 10 mice
D1 Correlation Time AIMs resolve vs ΔF/F returns to baseline	S3d	Pearson	8 mice x 4 timepoints	-	0.8803	-	-	N = 10 mice
D2 Correlation AIMs vs ΔF/F for LID onset	S3f	Pearson	9 mice x 5 timepoints	-	0.0011 $R2=0.22$	-	-	N = 10 mice
D2 Correlation AIMs vs ΔF/F for LID offset	S3g	Pearson	9 mice x 4 timepoints	-	0.0350 $R2=0.12$	-	-	N = 10 mice
D2 Correlation Time AIMs resolve vs ΔF/F returns to baseline	S3h	Pearson	9 mice x 4 timepoints	-	0.0018 $R2=0.25$	-	-	N = 10 mice
Number of c-Fos+ cells	3b	Kruskal-Wallis	Day 1: 4<br>Day 4: 5<br>Week 4: 5	-	0.8626	-	-	N = 5 mice/group
Number of c-Fos+/tdTom+ cells	3c	Kruskal-Wallis	Day 1: 4<br>Day 4: 5<br>Week 4: 5	-	0.2683	-	-	N = 5 mice/group
ΔFR of putative D1 MSN	3g	Kruskal-Wallis	Day 1: 10<br>Day 4: 10<br>Week 4: 14	Day 1: 55<br>Day 4: 48<br>Week 4: 157	0.0009	Day 1 vs day 4<br>Day 1 vs week 4	0.0179<br>0.0007	N = 5 mice/group<br>n = 20 units/group
ΔFR of putative D2 MSN	3j	Kruskal-Wallis	Day 1: 10<br>Day 4: 10<br>Week 4: 14	Day 1: 17<br>Day 4: 29<br>Week 4: 47	0.9213	-	-	N = 5 mice/group<br>n = 20 units/group
Peak GRAB-DA2h	4e	RM one-way ANOVA	8	-	0.3776	-	-	N = 10 mice
D1 mEPSC frequency	5b	Kruskal-Wallis	no LD: 11<br>day 4: 6<br>week 4: 6	no LD: 30<br>day 4: 22<br>week 4: 24	<0.0001	no LD vs day 4<br>no LD vs week 4	0.0141<br><0.0001	N = 5 mice/group<br>n = 20 cells/group
D1 mEPSC amplitude	5c	Kruskal-Wallis	no LD: 11<br>day 4: 6<br>week 4: 6	no LD: 30<br>day 4: 22<br>week 4: 24	0.0158	no LD vs week 4	0.0150	N = 5 mice/group<br>n = 20 cells/group
D2 mEPSC frequency	5e	Kruskal-Wallis	no LD: 10<br>day 4: 6<br>week 4: 6	no LD: 32<br>day 4: 20<br>week 4: 25	0.5896	-	-	N = 5 mice/group<br>n = 20 cells/group
D2 mEPSC	5f	Kruskal-	no LD: 10	no LD: 32	0.5135	-	-	N = 5

amplitude		Wallis	day 4: 6<br>week 4: 6	day 4: 20<br>week 4: 25				mice/group<br>n = 20<br>cells/group
D1 responses to depolarizing currents	6b	Two-way ANOVA	no LD: 9<br>day 4: 6<br>week 4: 6	no LD: 22<br>day 4: 13<br>week 4: 15	Treatment factor: 0.0001<br>Interaction : <0.0001	No LD vs week 4 (200, 300, 400, 500 pA)<br>Day 4 vs week 4 (300, 400, 500 pA)	<0.05<br><0.05	N = 5<br>mice/group<br>n = 15<br>cells/group
D1 rheobase	6c	Kruskal-Wallis	no LD: 9<br>day 4: 6<br>week 4: 6	no LD: 22<br>day 4: 13<br>week 4: 15	<0.0001	No LD vs week 4<br>Day 4 vs week 4	<0.0001<br>0.0253	N = 5<br>mice/group<br>n = 15<br>cells/group
D1 action potential threshold	6e	Kruskal-Wallis	no LD: 9<br>day 4: 6<br>week 4: 6	no LD: 21<br>day 4: 13<br>week 4: 15	0.0578	-	-	N = 5<br>mice/group<br>n = 15<br>cells/group
D1 AHP	6f	Kruskal-Wallis	no LD: 9<br>day 4: 6<br>week 4: 6	no LD: 21<br>day 4: 13<br>week 4: 15	<0.0001	No LD vs week 4<br>Day 4 vs week 4	<0.0001<br>0.0064	N = 5<br>mice/group<br>n = 15<br>cells/group
D1 half width	6g	Kruskal-Wallis	no LD: 9<br>day 4: 6<br>week 4: 6	no LD: 21<br>day 4: 12<br>week 4: 15	0.0021	No LD vs week 4	0.0013	N = 5<br>mice/group<br>n = 15<br>cells/group
D1 resting membrane potential	6h	Kruskal-Wallis	no LD: 9<br>day 4: 6<br>week 4: 6	no LD: 22<br>day 4: 13<br>week 4: 15	0.7714	-	-	N = 5<br>mice/group<br>n = 15<br>cells/group
D1 subthreshold voltage deflection	6i	Two-way ANOVA	no LD: 9<br>day 4: 6<br>week 4: 6	no LD: 22<br>day 4: 13<br>week 4: 15	interaction : <0.0001	No LD vs week 4 (at 100pA)	0.0211	N = 5<br>mice/group<br>n = 15<br>cells/group
D2 responses to depolarizing currents	S4b	Two-way ANOVA	no LD: 11<br>day 4: 6<br>week 4: 5	no LD: 24<br>day 4: 13<br>week 4: 17	Treatment factor: 0.1264	-	-	N = 5<br>mice/group<br>n = 15<br>cells/group
D2 rheobase	S4c	Kruskal-Wallis	no LD: 11<br>day 4: 6<br>week 4: 5	no LD: 24<br>day 4: 13<br>week 4: 17	0.3731	-	-	N = 5<br>mice/group<br>n = 15<br>cells/group
D2 action potential threshold	S4e	Kruskal-Wallis	no LD: 11<br>day 4: 6<br>week 4: 5	no LD: 23<br>day 4: 12<br>week 4: 17	0.6441	-	-	N = 5<br>mice/group<br>n = 15<br>cells/group
D2 AHP	S4f	Kruskal-Wallis	no LD: 11<br>day 4: 6<br>week 4: 5	no LD: 23<br>day 4: 12<br>week 4: 17	0.8176	-	-	N = 5<br>mice/group<br>n = 15<br>cells/group
D2 half width	S4g	Kruskal-Wallis	no LD: 11<br>day 4: 6<br>week 4: 5	no LD: 22<br>day 4: 11<br>week 4: 17	0.2539	-	-	N = 5<br>mice/group<br>n = 15<br>cells/group
D2 resting membrane potential	S4h	Kruskal-Wallis	no LD: 11<br>day 4: 6<br>week 4: 5	no LD: 24<br>day 4: 13<br>week 4: 17	0.7945	-	-	N = 5<br>mice/group<br>n = 15<br>cells/group
D2 subthreshold voltage deflection	S4i	Two-way ANOVA	no LD: 11<br>day 4: 6<br>week 4: 5	no LD: 24<br>day 4: 13<br>week 4: 17	interaction : 0.3247	-	-	N = 5<br>mice/group<br>n = 15<br>cells/group
1μM DA-evoked GIRK current	7d	Kruskal-Wallis	no LD: 7<br>day 4: 7<br>week 4: 8	no LD: 16<br>day 4: 15<br>week 4: 14	0.1274	-	-	N = 5<br>mice/group<br>n = 12<br>cells/group

100μM DA-evoked GIRK current	7e	Kruskal-Wallis	no LD: 7<br>day 4: 7<br>week 4: 8	no LD: 16<br>day 4: 15<br>week 4: 14	0.5348	-	-	N = 5 mice/group<br>n = 12 cells/group
DA-evoked GIRK current ratio	7f	Kruskal-Wallis	no LD: 7<br>day 4: 7<br>week 4: 8	no LD: 16<br>day 4: 15<br>week 4: 14	0.01	No LD vs Day 4<br>No LD vs Week 4<br>Day 4 vs Week 4	>0.9999<br>0.0098<br>0.0879	N = 5 mice/group<br>n = 12 cells/group

AIM: Abnormal Involuntary Movements
AUC: Area Under the Curve
LID: Levodopa-Induced Dyskinesia
FR: Firing Rate
mEPSC: miniature excitatory postsynaptic current
AHP: afterhyperpolarization
DA: dopamine
